# Extended study on atomic featurization in graph neural networks for molecular property prediction

**DOI:** 10.1186/s13321-023-00751-7

**Published:** 2023-09-19

**Authors:** Agnieszka Wojtuch, Tomasz Danel, Sabina Podlewska, Łukasz Maziarka

**Affiliations:** 1 Faculty of Mathematics and Computer Science, Jagiellonian University, Łojasiewicza 6, 30-348 Kraków, Poland; 2 Maj Institute of Pharmacology, Polish Academy of Sciences, Smętna 12, 31-343 Kraków, Poland

**Keywords:** Molecular property prediction, Atom featurization, Compound representation, Molecular graph, Graph neural networks

## Abstract

Graph neural networks have recently become a standard method for analyzing chemical compounds. In the field of molecular property prediction, the emphasis is now on designing new model architectures, and the importance of atom featurization is oftentimes belittled. When contrasting two graph neural networks, the use of different representations possibly leads to incorrect attribution of the results solely to the network architecture. To better understand this issue, we compare multiple atom representations by evaluating them on the prediction of free energy, solubility, and metabolic stability using graph convolutional networks. We discover that the choice of atom representation has a significant impact on model performance and that the optimal subset of features is task-specific. Additional experiments involving more sophisticated architectures, including graph transformers, support these findings. Moreover, we demonstrate that some commonly used atom features, such as the number of neighbors or the number of hydrogens, can be easily predicted using only information about bonds and atom type, yet their explicit inclusion in the representation has a positive impact on model performance. Finally, we explain the predictions of the best-performing models to better understand how they utilize the available atomic features.

## Introduction

Graph neural networks are widely used for predicting molecular properties. The interest in graph-based models has increased since they were shown to achieve competitive results and often outperform models based on molecular fingerprints [1–4]. As a result, new models for this purpose have been proposed [5–10].

Since the beginning, the main focus of the deep learning community has been on developing better machinery for processing graph data. For instance Veličković et al. [11] introduce the attention mechanism for graph neural networks, Li *et al.* [12] introduce a dummy super node—an artificial node connected to all nodes in the graph that is responsible for learning graph-level representation, and Ryu et al. [13] propose gated skip-connections—connections which omit several layers and are equipped with a forgetting mechanism that allows training networks with more convolutional layers. Meanwhile, the introduction of Transformer [14] with its remarkable triumphs in several domains [15, 16] inspired its adaptation to graph data [17, 18].

**Fig. 1:**
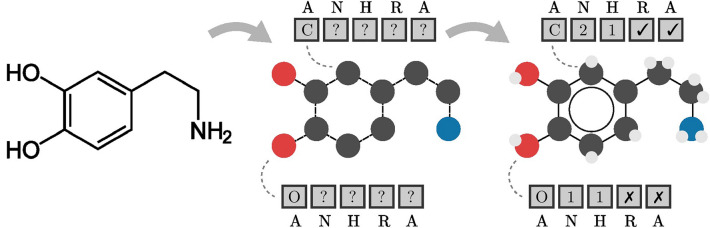
The information gain from using atomic features. Compound structure (left) can be encoded as a molecular graph in which atoms are nodes and bonds are edges. Atomic features are assigned to each node, and at least atom types (A) are required to identify atoms (middle). Bond identification (e.g. bond order) can be implicitly encoded in atomic features by providing information about the number of heavy neighbors (N) and implicit hydrogens (H). Other features such as inclusion in rings (R) or aromatic rings (A) can help graph models in finding relevant patterns (right)

Authors of new methods often neglect the impact of the used atomic representation. Therefore, atoms are represented differently for each new graph-based model, leading to unfair attribution of the results solely to the processing methods developed. This contrasts with many earlier works that used only a handful of well-established representations, such as ECFP [19] or KRFP [20], for benchmarking classical (non-neural) machine learning methods [21, 22]. The use of these fingerprints has become widespread, facilitating the comparison of various methods. It is crucial that while the existence of dataset benchmarks such as MoleculeNet [21] is fundamental for a fair comparison between different models, it does not solve the issues arising from the lack of standardization of the input representations. Arguably, models trained on different input representations have access to different information, just like models trained on different samples.

In Fig. 1, we present how the information available to a graph model changes based on the choice of atomic features. A compound structure (left) can be encoded as a molecular graph in which atoms are nodes and bonds are edges. The representation in the middle uses only information about the atom types. As a result, a graph model has access to information about which atoms are connected (shown with dashed lines) and what their types are (represented with colors). However, this model lacks information about bond order or the number of attached hydrogens which is available when more atomic features are used (right).

There is a need for a systematic comparison of graph representations, which seems independent of the choice of architecture. In this work, we focus on atomic features and examine how they affect model performance. We investigate which features are more useful than others and how models utilize them when making predictions.

Our contributions can be summarized as follows:

- We provide a comprehensive list of atom and bond features widely used in molecular graph representations for graph neural networks.
- We qualitatively and quantitatively evaluate the importance of atomic features by comparing performance of graph convolutional neural networks trained with twelve hand-crafted feature combinations and four combinations found in related literature.
- We demonstrate that the choice of atomic features is task-dependent. By explaining model predictions, we confirm that the importance of certain features correlates with their distribution in the dataset. As a result, removing scarce or redundant atomic features like formal charges or aromaticity can improve performance significantly.
- Finally, we confirm that the findings described above hold for more sophisticated models, including graph transformers, but the optimal selection of atomic features is model-dependent.

The code for the experiments is available online https://github.com/gmum/graph-representations.

### Related work

The two main components of molecular property prediction are the representation of chemical compounds and the model used to calculate the property values. The classical machine learning methods that were used to find the relationship between the chemical structure of molecules and their properties used simple 1D molecular descriptors, e.g. lipophilicity or electron density, to predict more complex molecular properties [23]. Shortly after, these descriptors were replaced by features derived from the structure of molecules.

A prominent example of structural compound descriptors is molecular fingerprints, which are mappings from chemical substructures to feature vectors of constant size. Vectors constructed in this way can become an input to machine learning models, such as random forests, support vector machines, or neural networks, to find quantitative structure–property relationships (QSPR) [24–26]. While substructural fingerprints became a standard for modeling various molecular properties, some methods employed specialized fingerprints adapted to the task at hand [27, 28].

ECFP fingerprint [19] is one of the most commonly used fingerprints in this setup [26, 29, 30]. It is calculated with an algorithm that uses a hash function to encode fragments that are present in the molecule. The crucial part of this encoding is the atomic representation, which makes the individual atoms in fragments distinguishable. As their representation, Rogers and Hahn [19] use the number of non-hydrogen neighbors, the valence minus the number of hydrogens, the atomic number, the atomic mass, the atomic charge, the number of attached hydrogens, and inclusion in rings.

Klekota-Roth fingerprint (KRFP) [20] is another representative example of a frequently used vector representation. Notably, it was designed by fragmentation of compounds selected based on their biological activity and pooling the resulting substructures. This demonstrates that careful choice of included information can lead to representations of general usefulness—KRFP was used to predict such properties as solubility [22], activity [31] or high-order electric properties [32].

With the development of recurrent neural networks for natural language processing, the textual SMILES [33] representation of a molecule became a common choice for both molecular property prediction [34–38] and molecule generation [39–42]. Nevertheless, this representation suffers from several drawbacks. SMILES encoding is not unique, which means that one molecule can be represented in several ways. The resulting strings may vary significantly, and it is impossible to determine if two SMILES encode the same molecule without translating them back to a molecular graph. This issue was only partially solved by the introduction of canonical SMILES [43]. Another disadvantage of SMILES is the limited information it can represent as it cannot be easily adapted for a downstream task.

Currently, graph representations of molecules are displacing molecular fingerprints, as graph neural networks can learn a molecular representation that is tailored to the prediction task. Graph convolutional neural networks (GCNs) [44–49] and, more recently, also graph transformers [17, 18, 50, 51] demonstrate outstanding results across numerous molecular property prediction tasks. Chuang et al. [52] discuss the crucial importance of molecular representations for tasks such as property prediction or generation of novel compounds. They highlight opportunities offered by representation learning, which is the ability of neural networks to learn internal representation specialized for the downstream task directly from data. The authors focus on the internal representation of neural networks, called latent space, and not on the input representation as in this work. However, the problem of input representation remains relevant, as it is the input representation that defines what information is available to the model and, in consequence, what information can be used to build the latent space.

The atomic representations diverge, beginning with the earliest works on GCNs. For example, Kearnes et al. [53] use atom types, chirality, formal and partial charge, ring sizes, hybridization, hydrogen bonding, and aromaticity. Gilmer et al. [6] use one-hot encoding of 5 atom types, hybridization, aromaticity, and whether an atom is an acceptor or donor, and add integer information about the atomic number and number of hydrogen neighbors. Coley et al. [5] encode only 10 most common atom types along with the number of atom heavy neighbors, the number of hydrogen neighbors, formal charge, aromaticity, and inclusion in a ring. Liu et al. [54] expand the one-hot representation to 22 most common atom types and add information about vdW and the covalent radius of the atom. However, they do not use information about atom neighborhood. Yang et al. [7] extend one-hot encoding to 100 dimensions and add information about atom’s chirality, atomic mass, hybridization, and number of bonds the atom is involved in. Moreover, some models also use bond representations. Commonly used bond features include bond order, stereochemistry, and information on whether a bond is conjugated or part of an aromatic system.

**Fig. 2:**
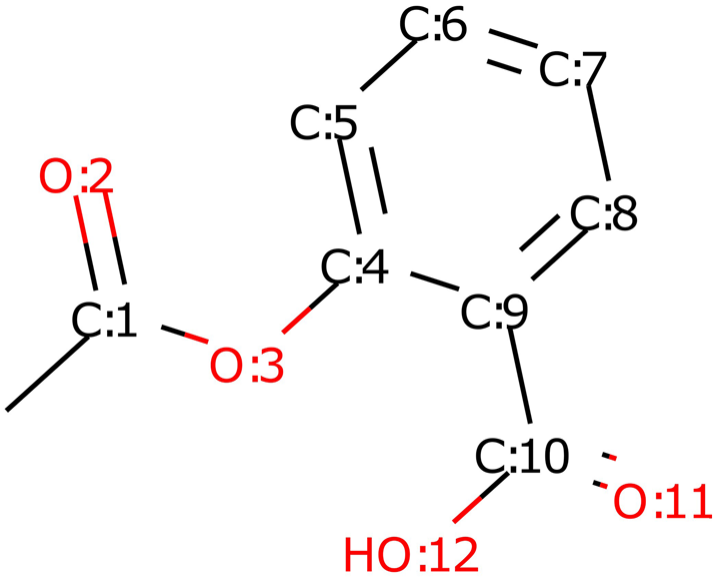
A 2D structure of aspirin. Graph topology cannot be used to approximate distances between atoms in space

Although molecular graphs encode more information than structural fingerprints, many important aspects of molecules are still lost. The graph topology does not encode 3D positions of atoms or their relative distances. This information cannot be approximated by the length of the shortest path between two atoms, which we illustrate using the compound shown in Fig. 2. As indicated in Table 1, the shortest path length is equal to 4 both between O:3 and O:11 and between O:3 and O:12; however, their relative distances in 3D space differ—they are equal to 2.68 and 4.17, respectively. At the same time, the distance between O:2 and O:11 is equal to 4.52 which is similar to that between O:3 and O:12, but in this case the shortest path length is equal to 6. Information about stereochemistry is another feature that is not preserved in graph topology. These deficiencies can be overcome by including 3D or stereo information among atomic features, which is another argument for the importance of atomic representation. However, global information about molecules, such as partition coefficient or polar surface area, which is available when using molecular descriptors, is still missing from graph representations. Attempts to make this information available to graph neural networks are already known in the literature [7, 49].

**Table 1:** The length of the shortest path and distance in 3D space between selected oxygen atoms of the molecule shown in Fig. 2

		Shortest path	Distance [Å]
O:3	O:11	4	2.68
O:3	O:12	4	4.17
O:2	O:11	6	4.52
O:2	O:12	6	6.24

A huge diversity of atomic representations makes it difficult to compare the performance of different models. One must take into consideration that the differences in performance might arise not only from the choices concerning the architecture but also from the representation being used. To the best of our knowledge, our research is the first comprehensive examination of atomic representations in graph neural networks. This study extends our preliminary work [55] that focused on a single graph architecture. We also provide an in-depth analysis of the atom feature importance across various molecular property prediction tasks.

## Atomic and bond features

Molecular graphs are attributed graphs in which atoms are vertices, and chemical bonds are edges. The vertices are attributed with atomic features that are transformed by graph layers to learn a more useful representation for a given prediction problem. Among the input atomic features are atomic symbols and the number of implicit hydrogens attached to the atom—hydrogens are often omitted in the molecular graphs, following the condensed line-angle formula convention from organic chemistry. Oftentimes, edges are attributed with bond features, e.g. bond orders. In the following, we describe the most common atomic and bond features.

### Atomic features

Atomic features encode local information about atoms and their surrounding. Atom attribution in graphs helps machine learning models to distinguish different chemical compounds that share the same carbon scaffold (graph topology), and furthermore it informs about local connectivity and 3D features.

*Atom types*: The most common feature included in atomic representations is the atom type, which is the chemical symbol of the atom. This is a crucial piece of information that allows one to differentiate between compounds that share the same graph topology. This information is included in the atomic featurization of almost all current molecular graph methods and is typically encoded with a one-hot vector. The set of encoded chemical elements is usually based on the input dataset, e.g. for organic druglike compounds, the set of encoded elements contains at least C, N, O, F, P, S, Cl, Br, and I. Hydrogens are often not included in the molecular graph (they are implicit), but it depends on the application.

*Number of hydrogens*: Since hydrogens are often implicit in molecular graphs, the number of hydrogens attached to an atom is typically included among the atomic features. This information also helps in inferring bond orders when they are not explicitly encoded in graph edges.

*Number of heavy neighbors*: Heavy neighbors are non-hydrogen atoms bonded to an atom. This information can be deduced from the molecular graph, but together with the number of hydrogens, it can substitute for the information about bond order and atom hybridization. Alternatively, *implicit valence* can be included to inform about the number of implicit hydrogens.

*Number of radical electrons*: Radicals are atoms with an unpaired valence electron, and the compounds containing them are reactive. Most datasets do not contain any radicals, so there is no need to encode the number of radical electrons. However, this information can be essential and complementary to the number of hydrogens and heavy neighbors, e.g. in chemical reaction datasets where radicals can occur.

*Charge*: Charges on atoms are encoded in different ways depending on the charge type. *Formal charges* are one-hot encoded integer numbers. On the other hand, *partial charges*, e.g. Gastgeiger charges or charges based on *electrostatic potential*, are represented with float numbers.

*Rings*: Inclusion in a ring can be marked as a binary value, by encoding the ring size in which the atom is present, or by encoding the number of rings that contain this atom, e.g. spiro atoms and bridges in bicyclic compounds are a part of multiple rings. *Aromaticity* of the rings is oftentimes encoded as well using a separate bit in the feature vector.

*Stereochemistry*: Orbital *hybridization* and *chirality* tags are among the most common features that explain the molecular geometry of the compound. They are encoded as one-hot vectors. For the chirality, the possible tags are R, S, and non-chiral. The set of encoded hybridizations is limited to the ones present in the input dataset. Furthermore, some representations include *van der Waals radii* or *atomic mass* to mark the space occupancy of the atom.

*Reactivity*: Sometimes atomic representations comprise features related to reactivity or interaction formation. For example, *acidic or basic* atoms can be marked, as well as *hydrogen bond acceptors and donors*.

### Bond features

Bond features can explicitly describe connections between atoms. Many graph neural networks omit bond attribution and use atomic features to deduce bond types in a molecular graph.

*Bond order*: Bond order is the most fundamental feature in resolving chemical structures. It is usually encoded as a one-hot vector, optionally comprising aromatic bonds.

*Conjugation*: Conjugated bonds, i.e. alternating single and double bonds, are often explicitly encoded in graph representation as they have specific chemical properties.

*Rings*: Information on whether a bond is included in a ring can be marked on a separate bit of the representation. *Aromaticity* can be also marked using an additional bit.

*Stereochemistry*: For double bonds, different isomeres can be encoded using *E-Z notation*, where E and Z are additional bits in the bond feature vector.

## Quantitative analysis

We represent atoms with six commonly used atomic features: one-hot encoded atom type with the following elements: B, C, N, O, F, P, S, Cl, Br, I, and additional bit for other atoms; the number of heavy (non-hydrogen) atom neighbors (0 to 5, one-hot encoded), the number of attached hydrogens (0 to 4, one-hot encoded), formal charge (single bit), inclusion in a ring (single bit), and aromaticity (single bit). We consider four representation groups:

- Using all the atomic features,
- Using only atom types,
- Using exactly one atomic feature besides atom type,
- Using all atomic features but one.

The details of all the representations are given in Table 2.

**Table 2:** Features included in each of the 12 atom representations

Name no.			A +					F-				
	F	A	A + N	A + H	A + C	A + R	A + A	F-N	F-H	F-C	F-R	F-A
	1	2	3	4	5	6	7	8	9	10	11	12
Atom type	$\checkmark$	$\checkmark$	$\checkmark$	$\checkmark$	$\checkmark$	$\checkmark$	$\checkmark$	$\checkmark$	$\checkmark$	$\checkmark$	$\checkmark$	$\checkmark$
Neighbors	$\checkmark$		$\checkmark$						$\checkmark$	$\checkmark$	$\checkmark$	$\checkmark$
Hydrogens	$\checkmark$			$\checkmark$				$\checkmark$		$\checkmark$	$\checkmark$	$\checkmark$
Formal charge	$\checkmark$				$\checkmark$			$\checkmark$	$\checkmark$		$\checkmark$	$\checkmark$
In a ring	$\checkmark$					$\checkmark$		$\checkmark$	$\checkmark$	$\checkmark$		$\checkmark$
Aromatic	$\checkmark$						$\checkmark$	$\checkmark$	$\checkmark$	$\checkmark$	$\checkmark$	

**Fig. 3:**
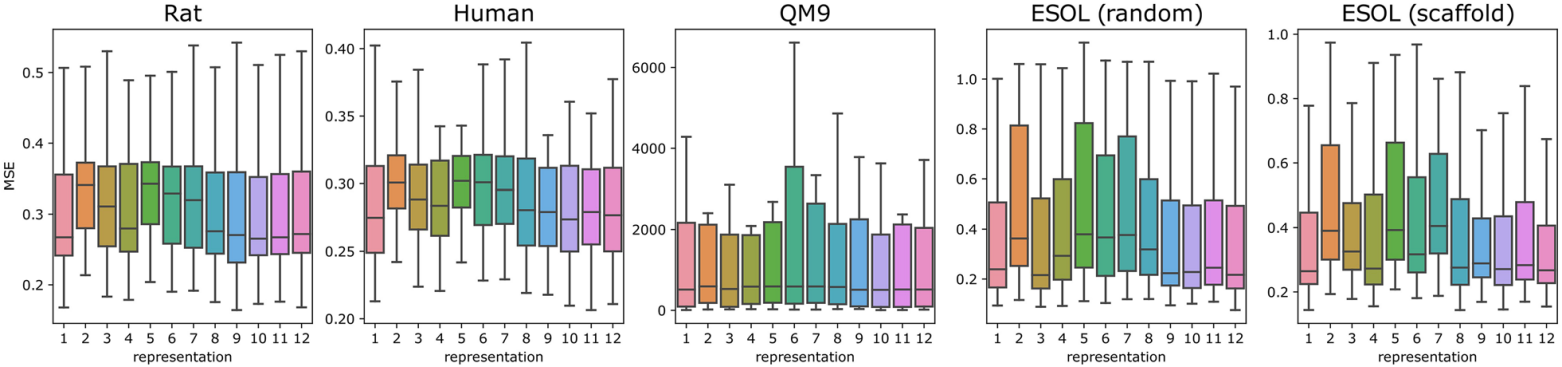
Distribution of mean square error on the test set of all models trained with the selected representation. Including more information in representation usually leads to better performance

For each representation, we train graph convolutional neural networks with 100 different architectures using mean squared error (MSE) loss. Moreover, we train two baseline models using ECFP fingerprints [19]. We report results on four datasets: water solubility dataset ESOL [56] which serves to compare results on data split randomly (ESOL (random)) and based on molecular scaffolds (ESOL (scaffold)), QM9 [57] – a dataset for predicting quantum properties, and two metabolic stability datasets, Human and Rat [24]. In Table 3, we summarize molecular properties predicted for each dataset, the dataset sizes, and property values ranges. Full details of the training procedure and datasets can be found in Section *Methods*.

**Table 3:** Description of the properties being predicted for each dataset along with dataset sizes and property values ranges

Dataset name	Predicted property	Range	Number of samples
Human	Metabolic stability $\hbox {T}_{1/2}$ (log h)	0, 4.8	3578
Rat	Metabolic stability $\hbox {T}_{1/2}$ (log h)	0, 4.5	1819
QM9	Atomization free energy at room temperature (eV)	− 714.6, − 117.8	134K
ESOL	Aqueous solubility (log M/L)	− 4.1, 2.2	1128

The property values were not normalized for the model training

We compare the average performance of the models trained with different representations in Fig. 3. Datasets and representations are on the x-axis and on the y-axis the distributions of mean square errors on the test set of all models trained with a given representation. One can see that the representation choice indeed has an influence on average model performance and that including more information in a representation usually leads to better performance (representations 1 and 8–12 perform better than representations 2–7).

While comparison between all models illustrates general tendencies, we are more interested in performance differences between only the best-performing models. We use performance on the validation set to choose a single best-performing architecture independently for each dataset and representation. The performance on the test set is averaged over different runs and training data (if applicable) and shown in Table 4. As expected, models trained with full representation (repr. F) consistently achieve good results, while models with information only about the atom type (repr. A) perform much worse. More generally, representations with more features (repr. F and F-) allow better performance than representations with limited information (repr. A and A+). Among all atomic representations, the ones with the lowest error use the full or almost full set of features. The only exception is ESOL (random) for which two models have comparable performance – one trained with representation F-H (no hydrogens) and the other with representation A+N (only atom types and neighbors).

**Table 4:** Average mean squared error on the test set of the best-performing model for each representation and dataset

Representation	Rat $\downarrow$	Human $\downarrow$	QM9 $\downarrow$	ESOL (random) $\downarrow$	ESOL (scaffold) $\downarrow$
F A	0.182 0.214	0.218 0.246	9.193 26.369	0.118 0.159	**0.166** 0.235
A + N A + H A + C A + R A + A	0.188 0.196 0.215 0.194 0.203	0.225 0.248 0.246 0.235 0.241	46.386 41.047 52.825 89.794 27.365	**0.113** 0.131 0.174 0.115 0.187	0.242 0.215 0.229 0.237 0.212
F-N F-H F-C F-R F-A	0.200 0.183 0.180 0.181 **0.178**	0.220 0.220 **0.213** 0.223 0.216	39.243 60.035 9.698 **8.278** 23.786	0.190 **0.113** 0.123 0.119 0.120	0.189 0.202 0.201 0.185 0.221
Tree-based baseline	0.207	0.235	699.125	0.432	0.801
XGBoost baseline	0.216	0.233	803.153	0.483	0.452

Two baselines based on the ECFP fingerprints are included. The best results are in bold. The error variance is below 0.001 for all datasets excluding QM9 and thus is not reported. Graph models perform better when trained with representations that include more features and usually outperform baseline models trained on traditional fingerprints

Comparison of models trained with information only about the atom type (repr. A) with models trained with a single additional feature (repr. A+) reveals that adding information about the number of heavy neighbors (repr. A+N) gives the largest increase in performance for three datasets (Rat, Human, and ESOL (random)). Moreover, adding information about the number of hydrogens (repr. A+H) significantly improves performance on ESOL (scaffold), though adding information about aromaticity (repr. A+A) produces even better results. Adding information about aromaticity also gives the best performance among models trained with representations A+ on QM9; however, not adding any additional information is even better in this case.

Comparison of models trained with full representation (repr. F) with models trained with representations that lack a single feature (repr. F-) confirms relevance of information about the number of heavy neighbors and aromaticity, and additionally demonstrates the relevance of hydrogens. When information about the number of neighbors is discarded (repr. F-N) there is a significant drop in performance for both metabolic stability datasets. In the case of hydrogens (repr. F-H), a significant drop can be seen for two datasets as well – Human and QM9. Importantly, in the case of Human, the largest drop in performance is seen when the discarded information concerns ring systems (repr. F-R). In the case of ESOL (random), the largest drop can be observed for models trained without information about formal charge (repr. F-C) and in the case of ESOL (scaffold) about aromaticity (repr. F-A).

**Fig. 4:**
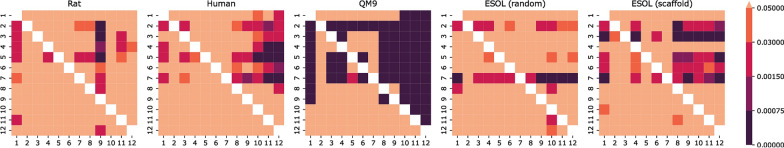
P-values of one-tailed Wilcoxon tests between the best models trained on each representation. The value in *i*-th row and *j*-th column corresponds to the alternative hypothesis saying that the median squared error of *i*-th representation is greater than the median of *j*-th representation (superior representations have darker columns, and inferior ones have darker rows). The darkest cells are statistically significant with Bonferroni correction

To systematically study the error distributions, we run Wilcoxon tests for a pairwise representation comparison. The p-values of the one-sided tests are plotted in Fig. 4. We observe that many representations are equivalent even before applying the Bonferroni correction (p $$\ge 0.05$$), e.g. for Rat, the lowest p-value is above the level of significance (p $$\ge 0.002$$ in a two-tailed Wilcoxon test, while the significant differences should be below 0.05/66 pairwise tests). The differences between representations are most apparent for QM9, which is the largest dataset in the comparison (p $$\le {\raise0.7ex\hbox{${0.05}$} \!\mathord{\left/ {\vphantom {{0.05} {66}}}\right.\kern-\nulldelimiterspace} \!\lower0.7ex\hbox{${66}$}}$$ in all two-tailed Wilcoxon tests in addition to the ones between representations A + N vs. A + C, A + C vs F−H, and F–C vs. F–R).

There are several patterns that can be observed in the heatmaps.

1. Representations with an almost full set of features are usually comparable with each other (bright area in the bottom right corner) and better than nearly empty feature vectors (dark area in the top right corner).
2. There are features that perform significantly worse than others when used alone, e.g. including only aromaticity (repr. 7) gives inferior results to using no atomic features in QM9 and ESOL with a random split. On the other hand, adding information about heavy neighbors (repr. 3) or hydrogens (repr. 4) yields the biggest performance boost across all datasets.
3. Removing features related to aromaticity (repr. 12), inclusion in a ring (repr. 11), and formal charges (repr. 10) can improve model quality, compared with the full representation (repr. 1).
4. Based on the dark cells in rows 2 and 7, we can conclude that representations A and A+A work significantly worse than other representations across all datasets.

## Feature prediction

After discovering that different atomic representations lead to significantly different performance of graph neural networks, we decided to test if some of the considered features can be predicted from the other ones. In other words, we test for the redundancy of atomic features. In these experiments, a graph neural network with only a subset of atomic features is trained to predict another subset of features. We formulate this task as node-level classification, i.e. the network predicts obscured features in each node. We use a random subset of 50 000 compounds from the ZINC database to perform this analysis. We randomly split the data into train and test sets using the 4:1 ratio.

**Table 5:** Mean node accuracy obtained for the classification of atomic features

Input features	Predicted features	Mean accuracy $\uparrow$ (%)
Atom type Atom type + #hydrogens Atom type + #hydrogens + in ring Atom type + #hydrogens + in ring + is aromatic	Number of neighbors Number of neighbors Number of neighbors Number of neighbors	99.99 100 100 100
Atom type Atom type + #neighbors Atom type + in ring Atom type + #neighbors + in ring Atom type + #neighbors + in ring + is aromatic	Number of hydrogens Number of hydrogens Number of hydrogens Number of hydrogens Number of hydrogens	91.76 92.67 93.08 93.57 98.00
Atom type Atom type + in ring	Is aromatic Is aromatic	92.87 94.64

Some atomic features are correlated with other properties or can be deterministically computed from other features and molecular graph topology. For example, the number of hydrogens for small organic molecules can be implicitly assumed based on the atom type and the number of neighboring heavy atoms. As can be seen in Table 5 the number of neighbors, the number of hydrogens and whether an atom is aromatic can be accurately predicted using only graph connections and atom types, which would suggest that these features are redundant and can be inferred from the data set. On the other hand, we saw in other experiments that including these features in representation improves model performance. We hypothesize that the input atom representation acts as an inductive bias in learning, which is especially helpful for small data sets.

**Table 6:** Prediction accuracy of inclusion in rings for different numbers of graph layers in the graph neural network

Number of graph layers	Mean accuracy $\uparrow$ (%)
1	88.41
2	94.12
3	97.01
4	98.00
5	98.72

Only atom types are used as input representation

In Table 6, we show the accuracy of prediction if an atom belongs to a ring system. The number of graph layers corresponds to the size of a receptive field in graph neural networks. We use only atom types as the input representation. A clear correlation between the number of graph layers and network ability to discover ring systems can be observed. Typically, for drug-like organic compounds, rings contain up to 8 atoms, and neural networks with 4 or more layers obtain almost perfect accuracy.

We conclude this section by stating that even though the examined atomic features can be predicted with high accuracy based on atom type and molecular graph topology alone, including them in representation is beneficial for model performance.

## Statistical analysis of explanations

In this section, we explain predictions of models trained with different representations to analyze which features they use and to what extent. We restrict our analysis to a single best-performing architecture for each dataset and representation. The averaged MSE of these models is reported in Table 4. We use GNNExplainer [58] to calculate how important each feature is for predicting a property of a given molecule.

**Fig. 5:**
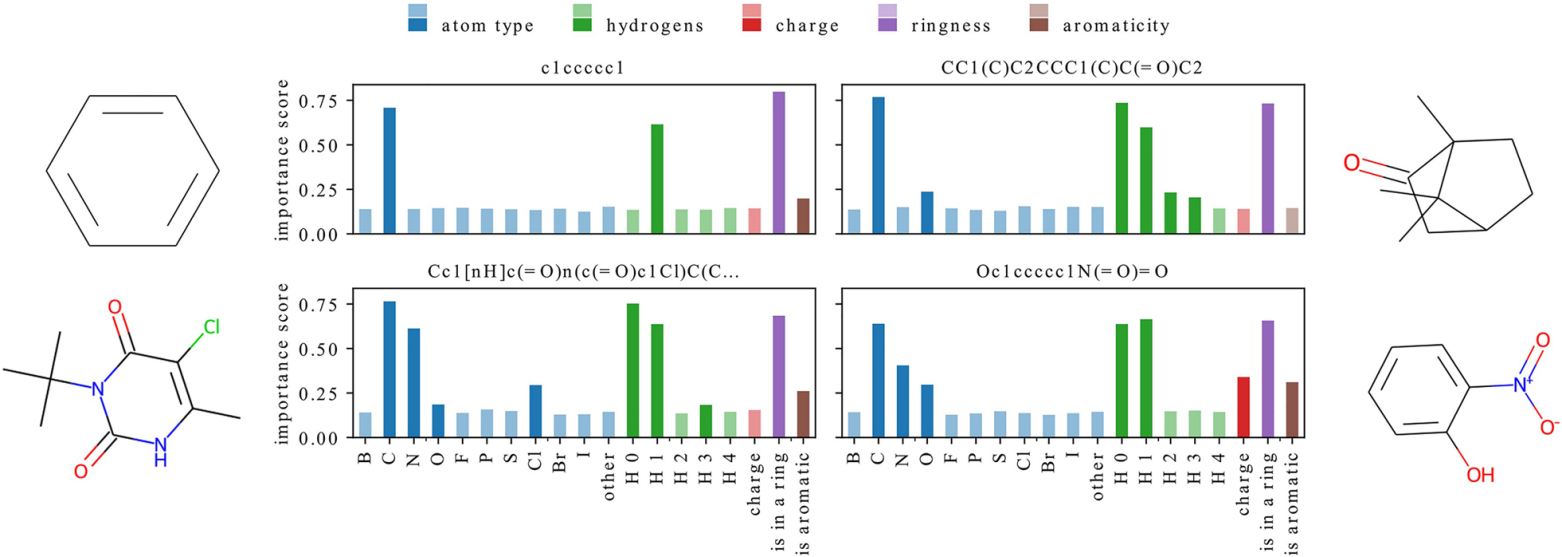
Feature importance scores for four predictions of a model trained with representation F-N on ESOL (scaffold). Importance score value (on y-axis) for each feature (on x-axis) is represented with a bar whose color denotes feature group and intensity denotes feature value – features with non-zero value for at least one atom in a molecule are shown in vibrant colors while features which are equal to zero for all atoms are shown in pale colors. A correlation between feature values and importance scores can be observed

**Table 7:** Absent-important ratio and present-ignored ratio for each dataset. Absent features are scarcely ever important (absent-important ratio is close to zero) while present features are typically used by models (present-ignored ratio has low values)

Dataset	Absent-important ratio	Present-ignored ratio
ESOL (random)	0.00	0.22
ESOL (scaffold)	0.01	0.18
Human	0.00	0.27
QM9	0.11	0.31
Rat	0.00	0.25

The analysis of Fig. 5 suggests that there seems to be a correlation between feature values and their importance scores. For all molecules, the bars of features with relatively high importance scores have vibrant colors, while most of the features of lesser importance are represented with pale-colored bars. We will call features with non-zero value for at least one atom in a molecule *present*, and features that are equal to zero for all atoms *absent*.

In Table 7, we measure the correlation between feature values and importance scores quantitatively. For each dataset, we calculate the number of present and absent features and the number of important and unimportant features in each of these groups. We say that a feature is important if its importance score is higher than the mean importance for the prediction. We define the absent-important ratio as the number of absent features that are important divided by the number of all absent features, and, analogously, the present-ignored ratio as the number of unimportant present features divided by the number of all present features. One can see that absent features are almost always unimportant, except for the QM9 dataset, where over 10% of absent features have importance scores higher than mean. At the same time, present features usually have high importance scores. Here again, QM9 models ignore present features most often – over 30% of the time. For models trained on other datasets, this value is between 18% (ESOL (scaffold)) and 27% (Human).

In Fig. 5, one can find exceptions to this general pattern – the importance of a feature is not always related to its presence in the analyzed structures. For example, both molecules on the left contain an aromatic ring but have low importance scores for this feature. Additionally, the top right molecule and both molecules at the bottom contain at least one oxygen atom, but this feature has a relatively high importance score only in the case of the molecule from the bottom right column. On the other hand, the presence of other atom types (such as C, N, or Cl) is usually related to an increased importance of the corresponding feature.

**Fig. 6:**
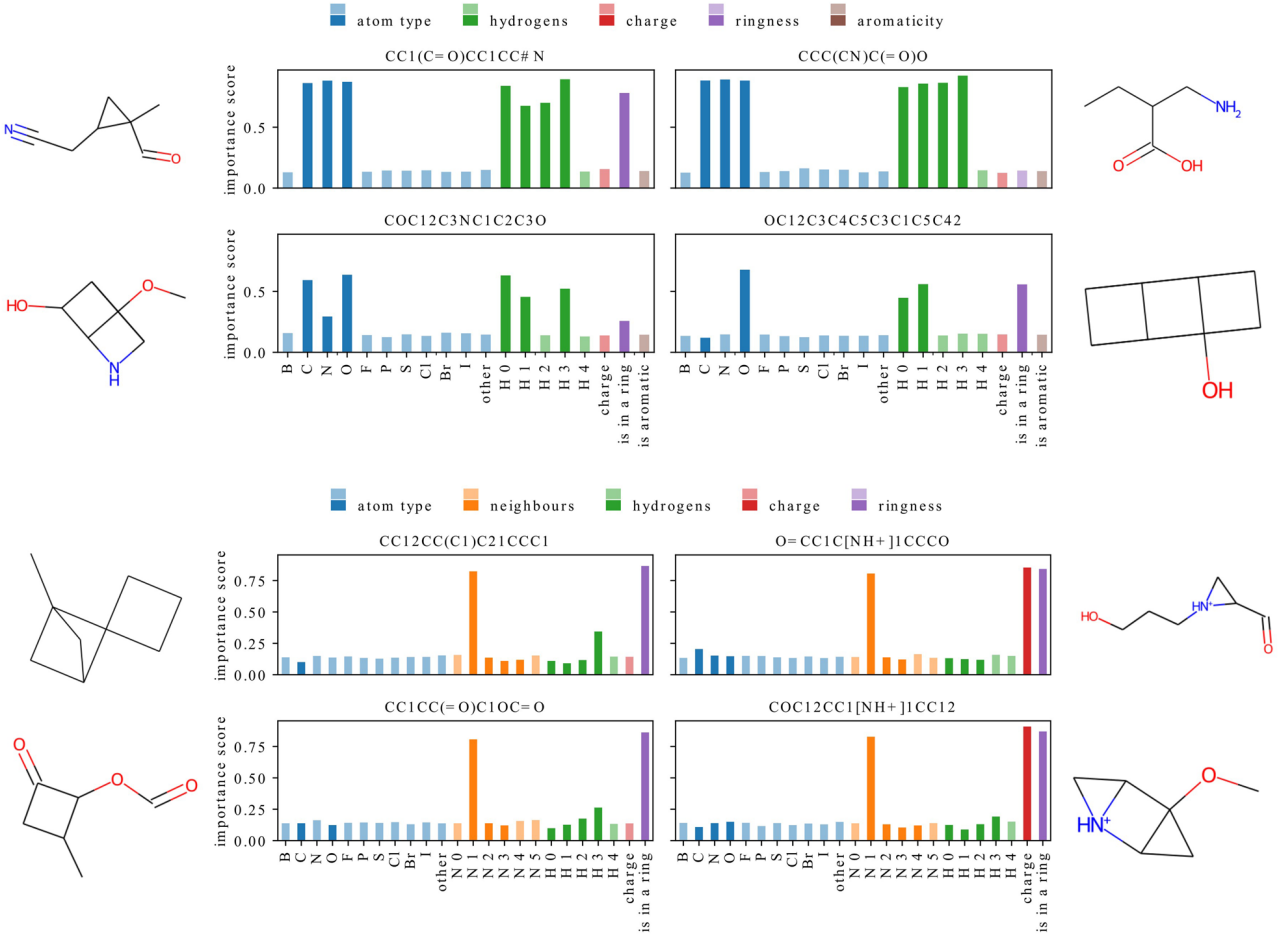
Feature importance scores for predictions of models trained on QM9 dataset with representation F-N (top) or representation F-A (bottom). A correlation between feature values and importance scores is not observed for all models

In Fig. 6, we show samples from the QM9 dataset for a model trained with representation F-N where the correlation between feature values and their importance can be observed (top) and for a model trained with representation F-A where it cannot be observed (bottom). In the top chart, the present features are indeed generally given more importance, while the absent features receive lower importance scores. This is not the case in the bottom picture where the only features with high importance are: having one heavy neighbor, charge, and being in a ring. The importance of the remaining features is limited regardless of their presence.

**Fig. 7:**
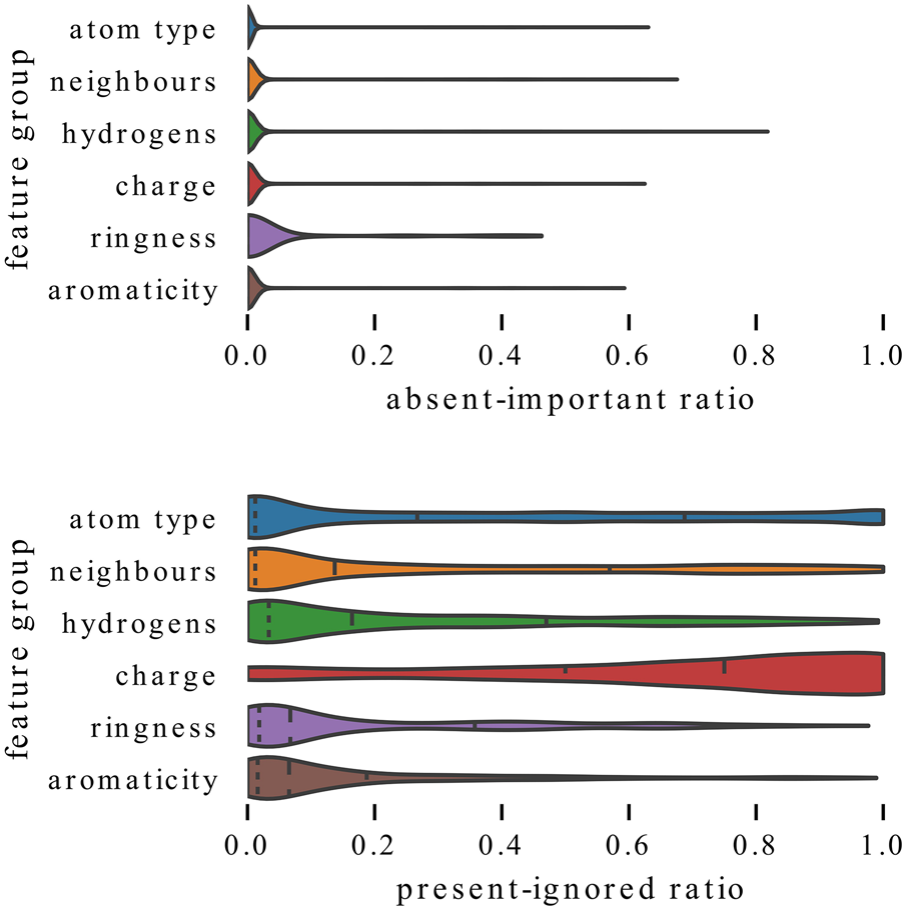
Distributions of absent-important ratios (top) and present-ignored ratios for each feature group (bottom). Absent features are consistently ignored while present features are typically important but show greater variability. Charge is often ignored even when present while the other features are used more frequently. Dashed lines show first quartile, median and third quartile

In Table 7, we presented absent-important ratios and present-ignored ratios calculated over predictions for all samples in each dataset. However, there can be significant differences between models with different representations or between different features. In Fig. 7, we show distributions of these ratios when calculated separately for each feature, model, and data part (train, validation, test or fold and test in the case of cross-validation). For brevity, the ratios of all features in the same group are shown as a single distribution. One can see that the absent-important ratio typically has values lower than 0.1 and slightly higher for inclusion in a ring than for other features. On the other hand, the present-ignored ratios have much higher values, sometimes reaching 1. This means that absent features are consistently ignored by models, while present features are usually important but in some cases ignored. Therefore, in the following analyses, we focus on the present-ignored ratios, which show higher variability. Moreover, in order to get more meaningful results, we only analyze ratios calculated on at least 20 samples and remove features which are not present in at least 100 samples in the training data because neural networks might not be able to learn to use features that are very rare.

**Fig. 8:**
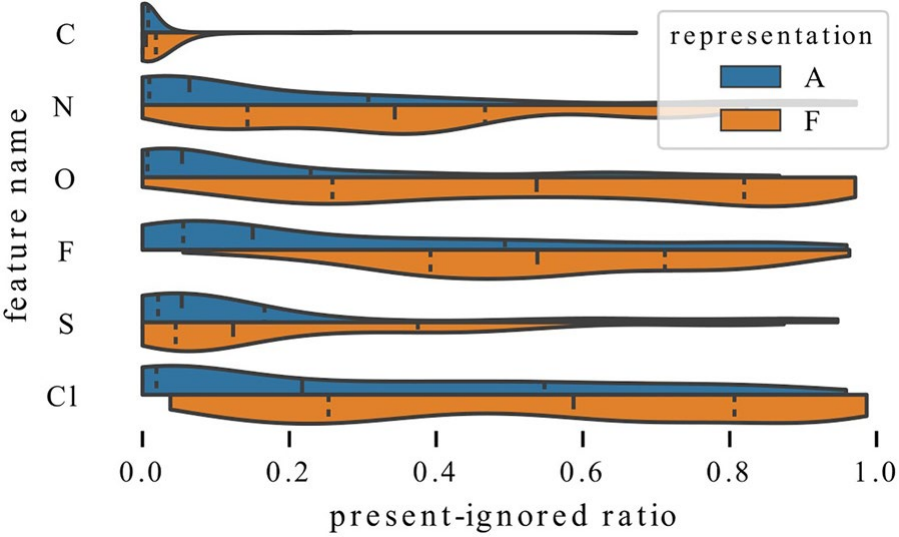
Comparison of exploitation of atom type features between models trained with representation F (full) and representation A (only atom types). Models that have access to additional information ignore atom types more often

We start by comparing models trained with representation F and representation A in terms of importance of each atom type – the distributions of present-ignored ratios are shown in Fig. 8. As expected, models trained with representation A strongly rely on atom types and ignore these features less often than models trained with full representation that can use plenty of other features.

**Fig. 9:**
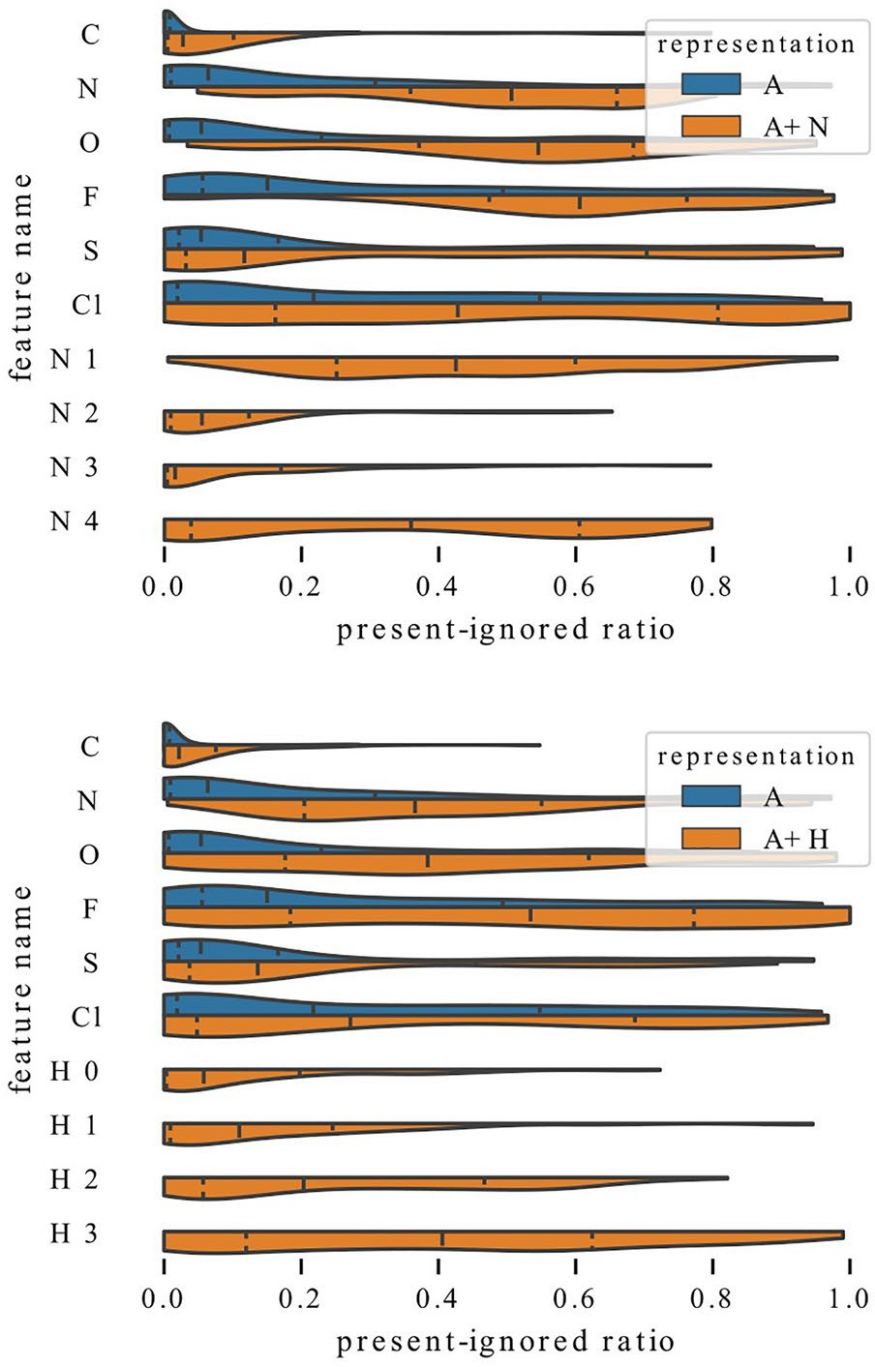
Influence of enriching representation with a single feature. Adding information about number of neighbors (top) or hydrogens (bottom) allows models to rely on atom types to a lesser extent

**Fig. 10:**
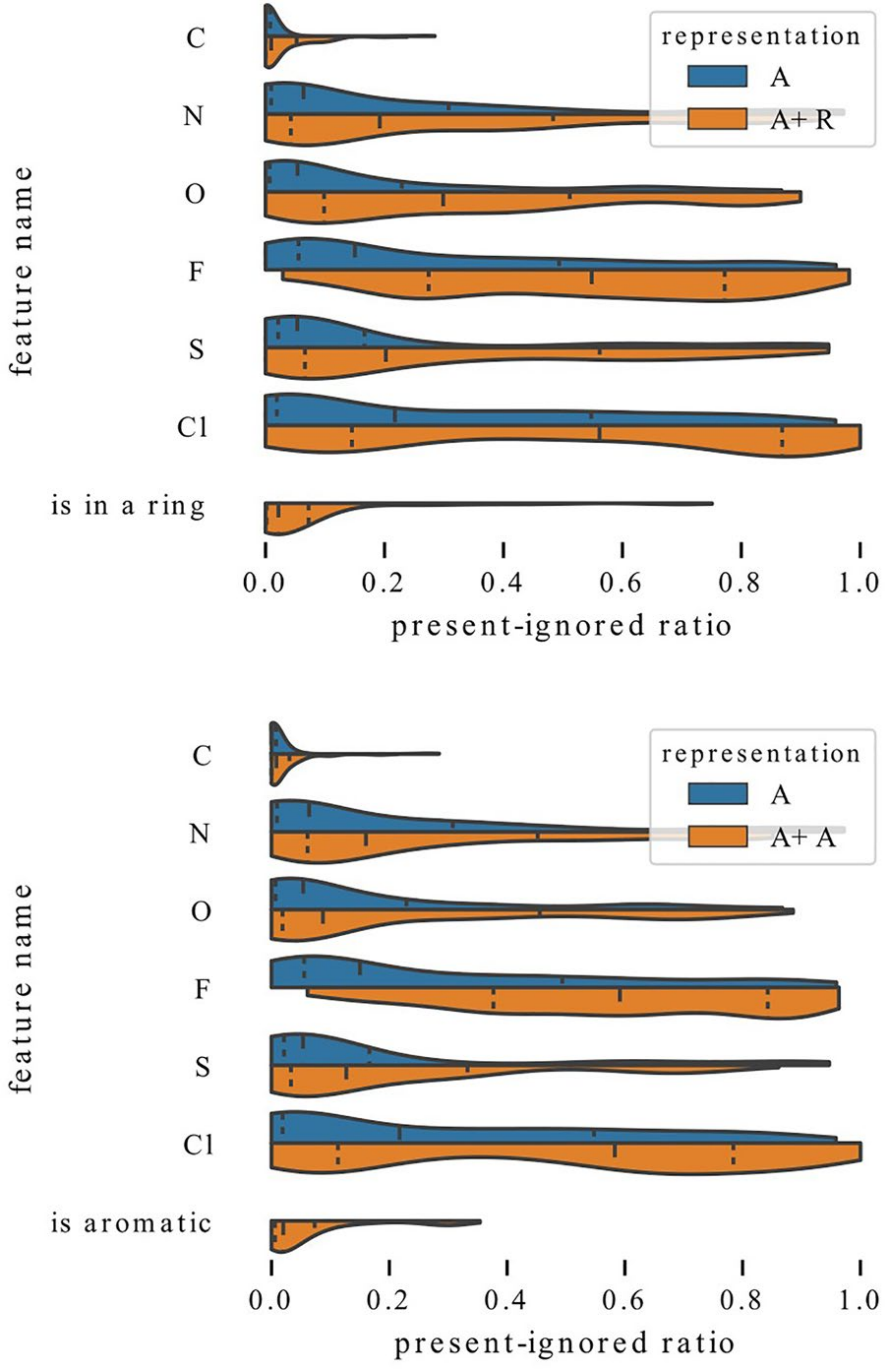
Influence of enriching representation with a single feature. Adding information whether atom is in a ring (top) or an aromatic ring (bottom) allows models to rely on atom types to a lesser extent

In Figs. 9, 10, we analyze the influence of adding a single feature group to a representation by comparing distributions of present-ignored ratios of representation A models with distributions for models trained with representations A+.

Examination of Fig. 9 reveals that adding information about the number of heavy neighbors or hydrogens lowers the average importance scores of all atom types. In both cases, this can be clearly seen for carbon, which is rarely ignored by any models. However, models that have access only to information about atom type rely on this feature more strongly. Distributions for nitrogen and oxygen are also visibly shifted towards higher values, both when the added information is the number of heavy neighbors and hydrogens. On the other hand, the distribution shift for sulfur is less emphasized. Some differences can also be observed. The distribution shift for fluorine and chlorine is much stronger when the added information is the number of heavy neighbors than when it is the number of hydrogens. This suggests that the former one might be more informative for graph models. The additional features are used to a various extent. For example features N 2 and N 3 (two or three heavy neighbors) have mean present-ignored ratios equal to 0.1 while feature N 1 (one neighbor) has an average ratio of 0.44 (note that visualizations show median, not mean value).

In Fig. 10, we visualize the change in distributions when the added information encodes inclusion in a ring or aromaticity. Again, it is visible that models that have access to more features rely on atom types to a lower extent, and the vital importance of carbon is confirmed. Here, the distribution shifts for nitrogen and oxygen are not as strong as in the case when adding information about the number of heavy neighbors or hydrogens, and for models trained with representation A+A it is quite subtle.

To summarize, the analysis of Figs. 9 and 10 demonstrates that information about the number of heavy neighbors, hydrogens, inclusion in a ring or aromaticity can partially substitute for information about the atom type, which is indicated by higher present-ignored ratios of models that have access to additional features. Moreover, information about whether an atom is a carbon is vital and rarely ignored, regardless of the representation.

**Fig. 11:**
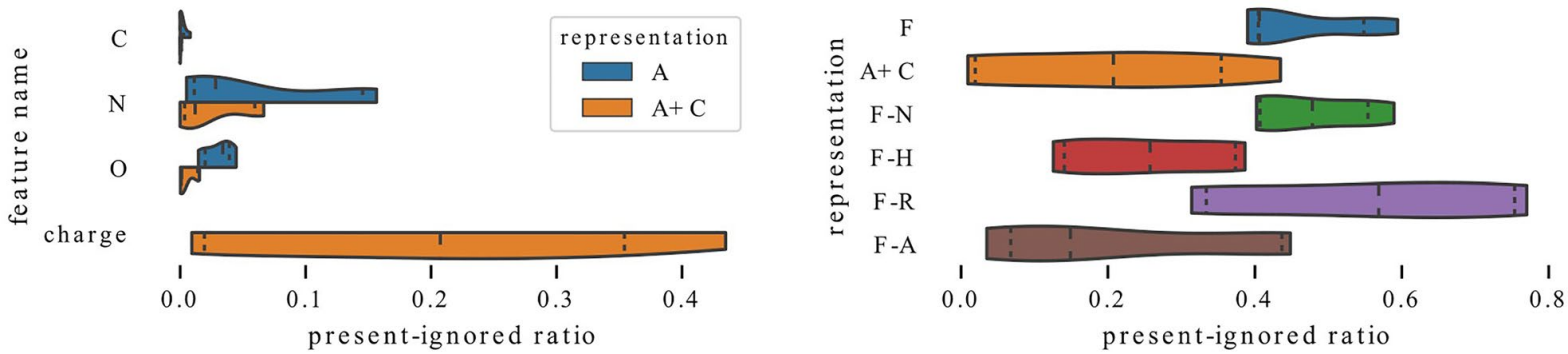
Exploitation of charge for QM9 models. Influence of enriching representation with information about charge (left) and exploitation of charge for models trained with different representations (right). Regardless of the representation, a high fraction of models ignores charge relatively often

On the other hand, information about charge is a poor substitute for atom type. In Fig. 11, we focus on charge and its usefulness for graph models. In this case, we restrict our analysis to QM9 models because the number of molecules with charge is too small in other datasets (less than 100 molecules). Moreover, we note that QM9 molecules consist of only carbon, nitrogen, oxygen and fluorine atoms; however, the number of molecules that contain fluorine in our training data is too small and we discard this feature. On the left, we compare models trained with information only about atom types with models which additionally have access to information about charge. The influence of charge is quite opposite to the influence of other features – models that have access to this feature rely on atom types even more strongly than models trained with representation A. At the same time, this feature is relatively often ignored, which can be seen in the right figure. While models for which charge is the only available feature besides atom type have an average present-ignored ratio equal to 0.2, for models trained with full representation this value increases to 0.46. Models trained with representation F-R ignore charge to the highest extent with average present-ignored ratio equal to 0.55. This is surprising because charge is a useful feature for predicting free energy. These findings suggest that charge is not a useful feature for graph models and its inclusion in the representation might be detrimental to the model performance. This is in line with the Wilcoxon analysis illustrated in Fig. 4 where representation A+C (no. 5) is inferior (has darker rows) for all datasets but ESOL (random).

**Fig. 12:**
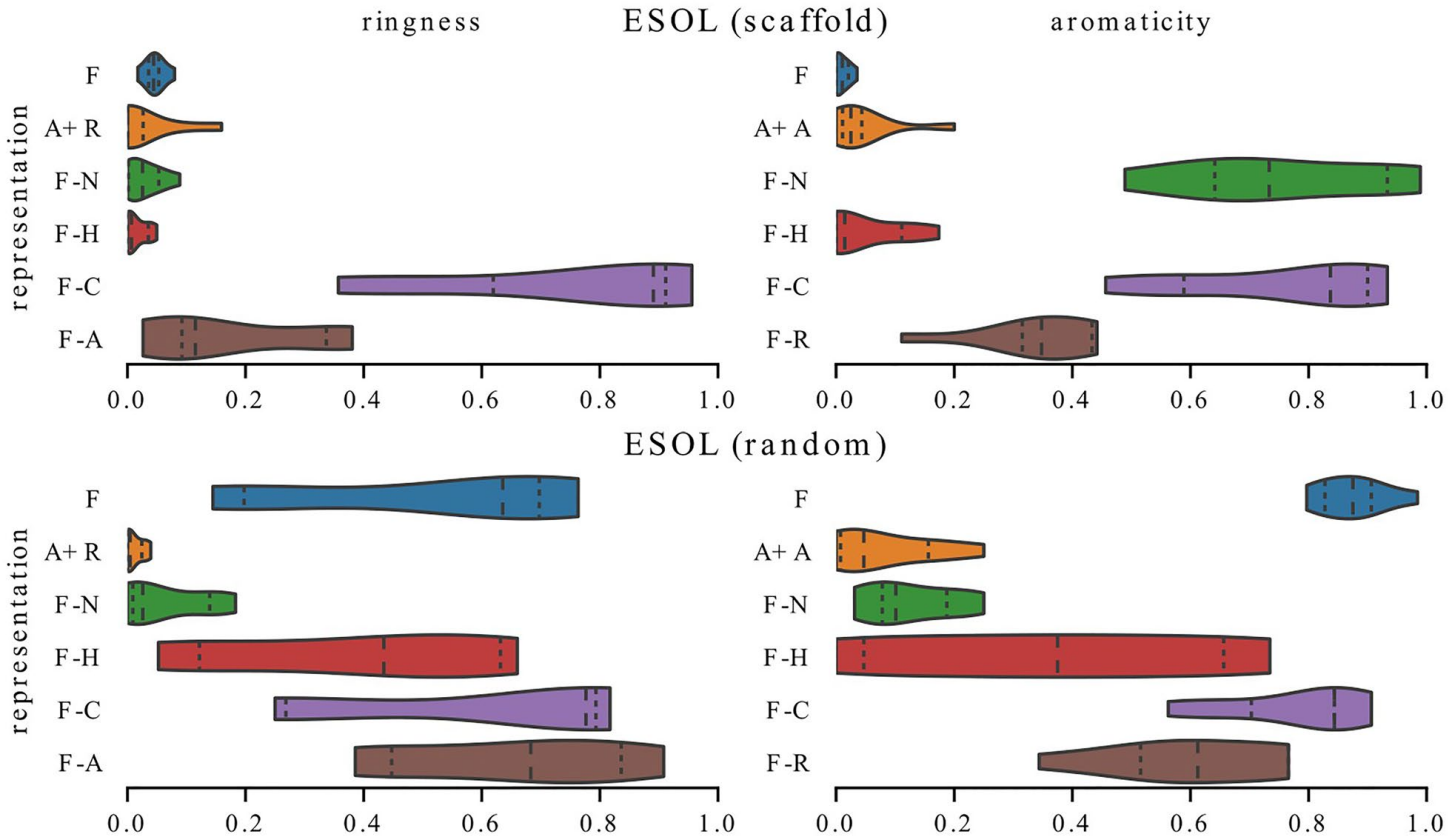
Exploitation of information about inclusion in a ring and aromaticity by ESOL models. These two features carry a similar information but are utilized differently

**Fig. 13:**
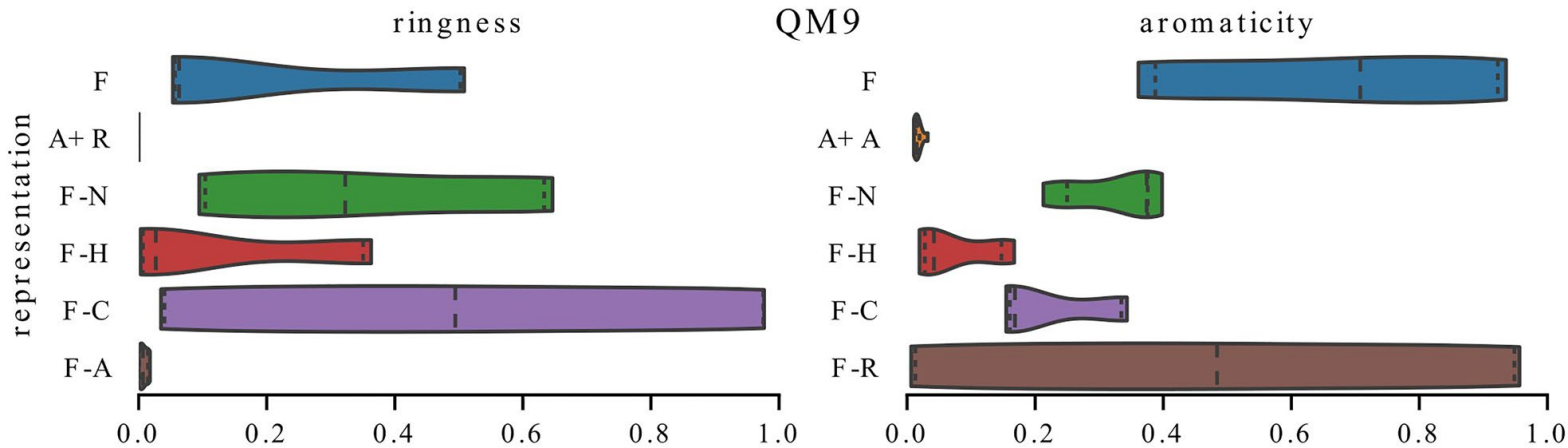
Exploitation of information about inclusion in a ring and aromaticity by QM9 models. These two features carry a similar information but are utilized differently

**Fig. 14:**
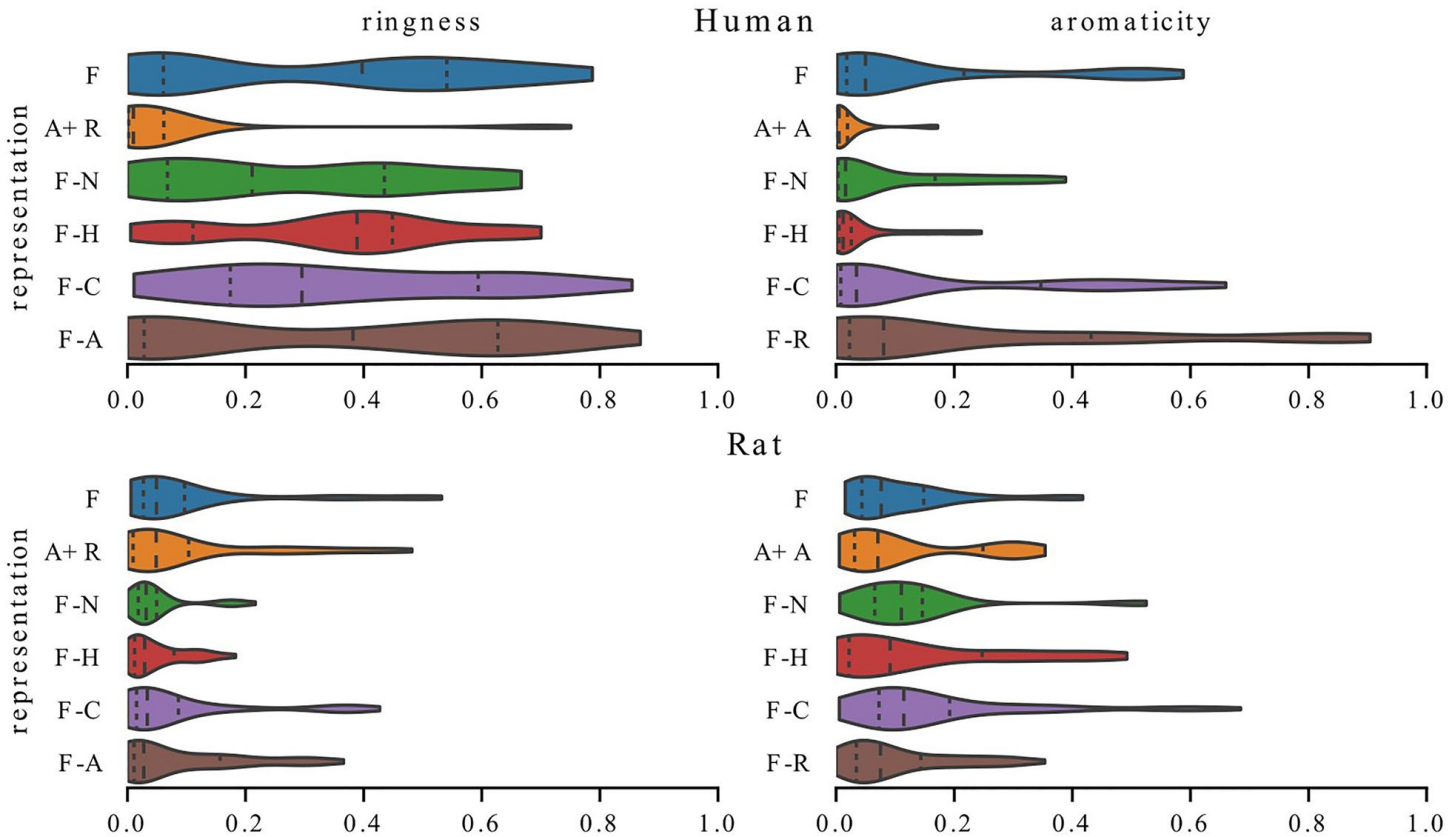
Exploitation of information about inclusion in a ring and aromaticity by models trained on metabolic stability datasets. Models trained on the same task (metabolic stability) may utilize these features to a different extent

Inclusion in a ring and aromaticity are highly correlated features. In Figs. 12, 13, 14, we compare their distributions of the present-ignored ratios across all datasets in the study and show that even though they carry similar information, they can be used in different ways.

In Fig. 12, we focus on models trained on the water solubility task. In most cases, features encoding inclusion in a ring and aromaticity are utilized similarly—distributions of present-ignored ratios for the corresponding models are alike. However, for ESOL (scaffold) models trained with representation F-N rely on ring information to a much higher extent than on aromaticity. For ESOL (random) and models trained on full representation, the situation is somewhat similar, which is indicated by a long tail of the ring distribution.

In Fig. 13, we focus on the QM9 dataset. The biggest difference can be seen for models trained without charge information which seem to prefer aromaticity. Interestingly, when this feature is dismissed, the models start to strongly rely on information about rings (compare distributions for representations F and F-A on the left plot); however, when information about rings is missing, the models do not necessarily replace it with information about aromaticity – distribution F-R in the right plot is widely spread.

In Fig. 14, we show distributions for metabolic stability datasets. One can see that Human models choose to ignore ring information more often than aromaticity, while for Rat models the differences are less emphasized and the preference is towards ring information.

**Fig. 15:**
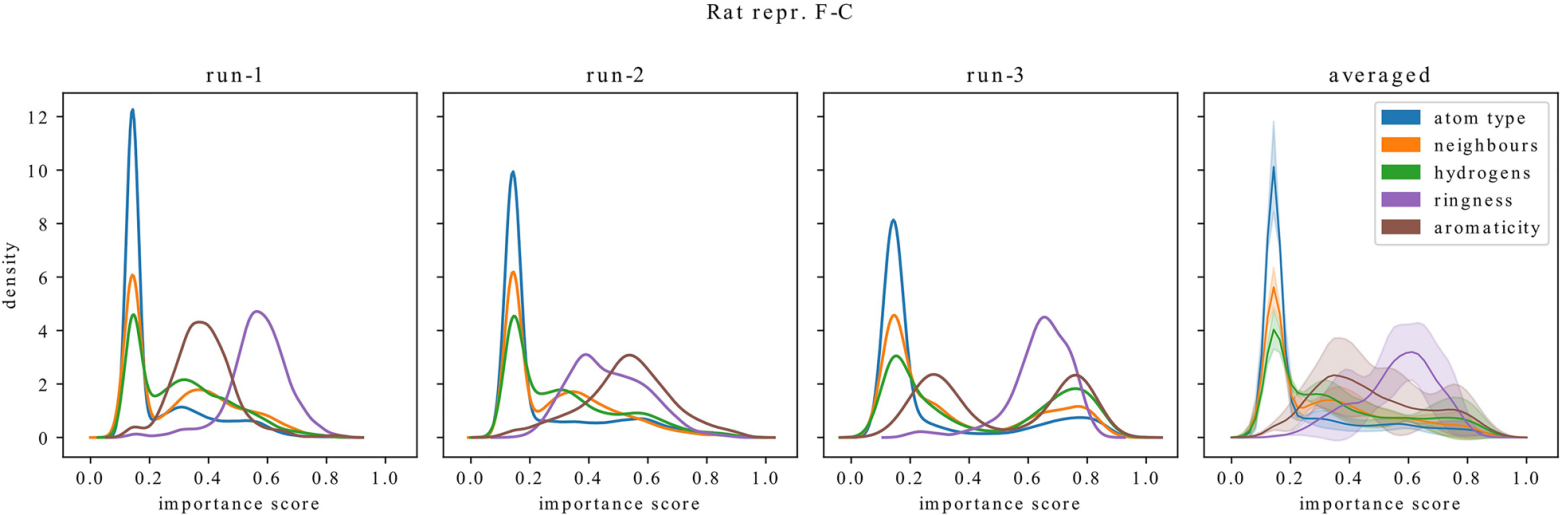
Distributions of feature importance scores for three GCN models trained on Rat dataset with representation F-C which differ only by weight initialization (run-1 - run-3). For each feature type, the mean of the corresponding distributions and the standard deviation is shown on the right (averaged). Models which differ only by initial weight values may show differences in their exploitation of information about inclusion in a ring and aromaticity while for other features the variability between models is lower

Interestingly, even when models share the same architecture, representation and training data (i.e. they differ only by random initialization of weights before training), there is a diversity in how information about inclusion in a ring and aromaticity is used. This is possible because neural networks that differ only by weight initialization can represent highly diverse functions [59]. We illustrate the differences between such models in Fig. 15 where we show distributions over importance scores for three models trained on the Rat dataset with representation F-C. One can see that the model on the left-hand side (run-1) relies on information about inclusion in a ring to a higher extent than on aromaticity. In the case of the model in the middle (run-2), the situation is reversed, although there is a larger overlap between these two distributions. For the model on the right-hand side (run-3), the feature importance scores for aromaticity form a bimodal distribution, which is not the case for the models on the left or in the middle. Importantly, the distributions for the remaining feature types (atom type, number of neighbors, and number of hydrogens) are more consistent – in the right-most plot of Fig. 15 (averaged), we present the mean and standard deviation for the distributions of each feature. The exact values of the present-ignored ratios averaged across the entire dataset for these models are given in Table 8 and confirm that there are differences in how information about inclusion in a ring and aromaticity is used.

**Table 8:** Mean present-ignored ratios for two features and test MSE calculated for Rat models trained on representation F-C which differ only by weight initialization

Run	Is aromatic	Is in a ring	Test MSE
Run-1	0.13	0.02	0.172
Run-2	0.05	0.08	0.192
Run-3	0.37	0.03	0.188

We conclude this section by stressing that features are used differently depending on the end-task; however, some general patterns emerge. Absent features are usually ignored, and it might be profitable to remove from representation any features that do not appear in the dataset often enough. Information whether an atom is a carbon is vital, while for other atom types, there is greater variability. The exact elements that should be encoded in a representation can be selected based on the training data. Charge is often ignored even for datasets that contain plenty of molecules with charge, whereas other features that we examined are more useful and can partially substitute for information about the atom type. Inclusion in a ring and aromaticity can be utilized by models in different ways, even though they carry similar information.

## Visualization of molecules with the highest prediction error

**Fig. 16:**
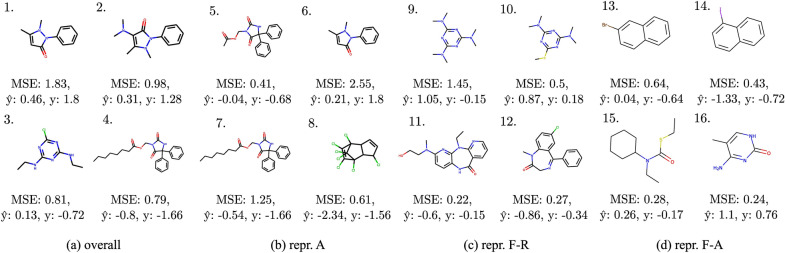
ESOL (scaffold) molecules for which prediction is particularly difficult for models trained with selected representations. Plots show compounds with the highest MSE in all representations (**a**), and MSE higher than in other representations (**b**–**d**); $\hat{y}$ is the average predicted value, and *y* is the true value (standardized)

In this section, we examine the molecules with the highest mean error depending on the representation used for training. To pick molecules that are especially difficult for models trained with a given representation, we calculate a margin between the mean error of models trained with this representation and the highest mean error of models trained with the remaining representations. To put it more precisely, for each compound, we calculate the predictions using the best model for each representation $\hat{y}_1,\dots ,\hat{y}_{12}$ and compare these predictions with the true label *y*. Next, we sort the compounds by the following value:

1 $$\begin{aligned} m_i = \max \left( 0, \varepsilon (y - \hat{y}_i) - \max _{\begin{array}{c} j=1,\dots ,12 \\ i\ne j \end{array}}\varepsilon (y-\hat{y}_j)\right) , \end{aligned}$$

where $\varepsilon :\mathbb {R}\rightarrow \mathbb {R_+}$ is an error function (e.g. MSE or MAE), and $m_i$ is the error margin of the compound for the *i*-th representation.

**Fig. 17:**
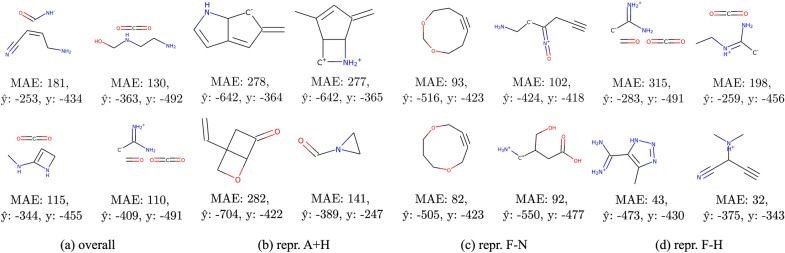
QM9 molecules for which prediction is particularly difficult for models trained with selected representations. Plots show compounds with the highest MAE in all representations (**a**) and MAE higher than in other representations (**b-d**); $\hat{y}$ is the average predicted value, and *y* is the true value (standardized)

In Fig. 16, we present molecules with the highest mean error of solubility prediction for all representations jointly and for three selected ones. Using only topological graph information and no atom features besides atom type produces similar structures to those that are on average worst predicted by all representations. For instance, the molecule with a long aliphatic chain (molecule 7) is predicted to be more soluble, probably because models with no additional atom features besides atom type cannot differentiate between saturated and unsaturated chains. Similarly, the compound with a cyclohexane ring (molecule 15) could be predicted to be more soluble due to the lack of aromaticity information—the aromatic counterpart of cyclohexane, a benzene, is more soluble in water. Furthermore, we note that models trained with the representation without information about ring inclusion (repr. F-R) often make mistakes for compounds with non-aromatic rings or nitrogens in rings.

Similar results for the QM9 dataset can be found in Fig. 17. In these selected representations, we again observe recurring patterns. For example, the representation without information about attached hydrogens results in poor predictions for compounds with nitrogen cations. Similarly, models trained with the representation missing the number of heavy neighbors obtain the highest error values for branched structures with carbocations or carbanions.

**Fig. 18:**
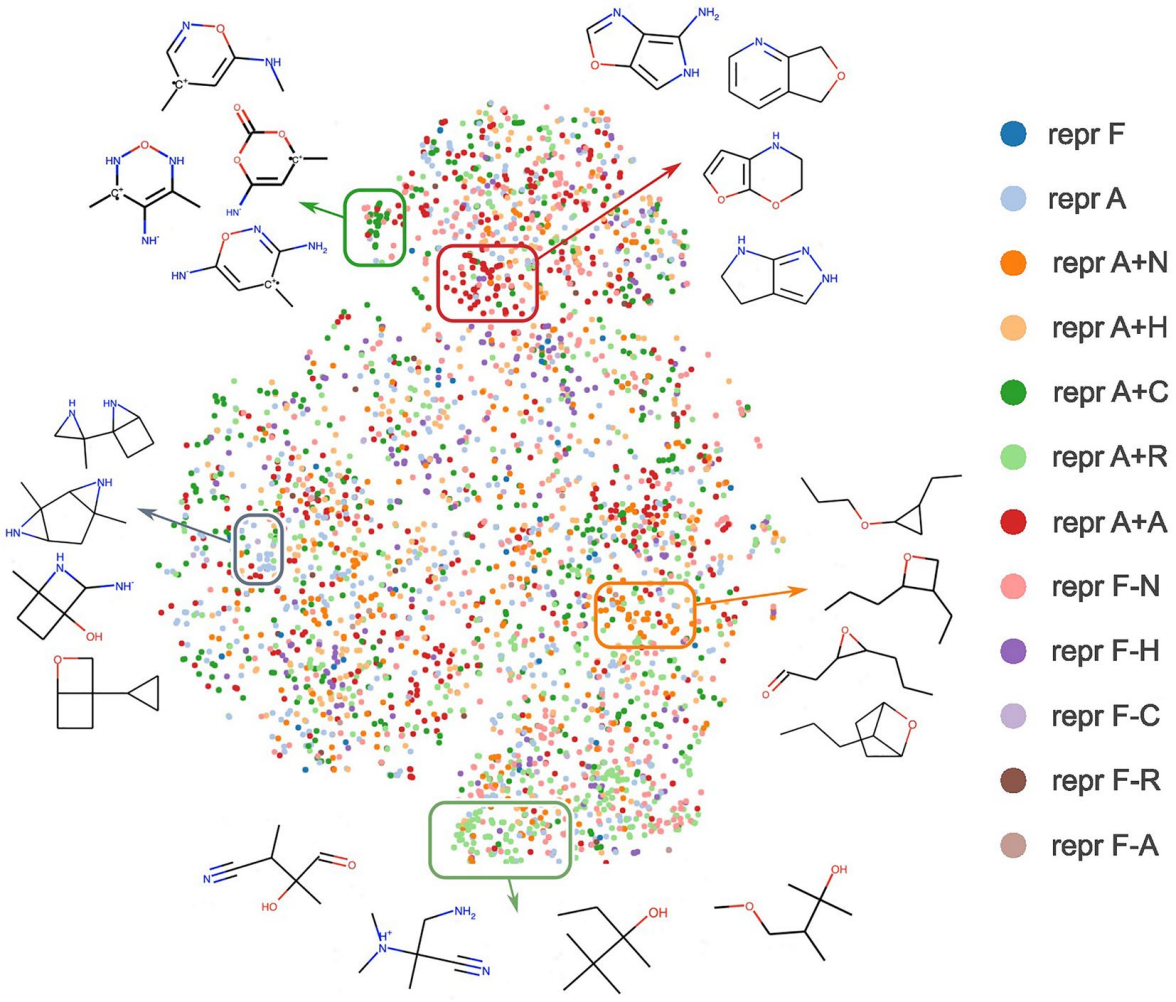
t-SNE map of QM9 compounds colored by the representation with the highest prediction error (MAE). The algorithm uses ECFP fingerprints and Tanimoto distance

To confirm that some representations are more prone to errors when certain patterns appear in the molecular structure, in Fig. 18 we present a t-SNE map of QM9 compounds. In the plot, each color corresponds to the representation with the highest prediction error.

We observe that small clusters of one color form in the t-SNE map. These clusters correspond to structural motifs that confuse models trained with a given representation. This observation suggests that some representations fail to predict certain structural patterns due to inductive biases. For example, representation A+R models tend to make errors for structures with many branches and no rings (depicted at the bottom of Fig. 18). Interestingly, representation A+C incorrectly predicts a group of compounds with a carbocation in a ring even though it contains information about the formal charge. A similar cluster corresponds to representation A+A that makes mistakes for predictions on fused bicyclic compounds that are partly aromatic.

## Discussion

In this study, we analyze the impact of atom representation on model performance and examine how selected features are utilized by models trained with different representations.

Our findings are based on experiments performed on datasets for different tasks and with different splits. We designed twelve representations that allow studying the impact of single atomic features in isolation and conducted additional experiments with selected representations previously used in literature (presented in the Appendix). The main experiments are performed using simple graph convolutional neural networks, and further experiments (presented in the Appendix) use advanced architectures that represent both GCNs (D-MPNN) and Transformer-based models (MAT), which are a distinctive family.

However, some limitations must be brought to light. First, we only explore the role of atom features and neglect the role of bond features, which currently are often used [5, 7, 49]. Second, we examine the performance on prediction of only three molecular properties and all tasks that we consider are regression. We do not include any classification tasks (e.g. prediction of toxicity [60] or bioactivity [61]) nor consider other important tasks such as de novo generation [41, 62], molecule optimization [63] or docking [64, 65]. Finally, we do not investigate multitask learning setting in which information about one task can be used for other tasks – incorporation of this additional knowledge may have an impact on the role of input features. Multitask learning has been shown to be a promising direction in QSPR [26].

Despite these limitations, we provide a comprehensive study on the impact of atomic features on the predictive power of models trained for molecular property prediction. We confirm that the choice of atomic representation influences model performance and recognize the necessity of taking it into consideration when comparing different models or creating benchmarks. Furthermore, we show that charge, which is a commonly used atom feature, might, in fact, be detrimental to model performance. Moreover, we reveal the correlation between feature values and their importance scores, which can serve as a rule of thumb for selecting atomic features for new tasks. Visualization of molecules with the highest prediction error indicates that mistakes committed by models trained with inadequate representations can be attributed to inductive biases present in these models. Finally, we expose dangers associated with an extensive search over atomic features (results presented in the Appendix).

## Conclusions

In this study, we establish that the careful selection of atom features used in the representation has a significant impact on the performance of graph models, including both standard graph convolutional neural networks and more advanced architectures (Appendix: Section *Supplementary experiments using advanced graph models*). Even though the optimal set of features is task-dependent, representations that have almost all the atomic features are generally comparable. Having said that, excluding features related to aromaticity, inclusion in a ring, and formal charges can improve model quality. On the other hand, including information about heavy neighbors or hydrogens gives the biggest performance boost across all datasets. Furthermore, we show that GCNs can accurately predict the number of heavy neighbors, hydrogens, inclusion in a ring, and whether an atom is aromatic, given only information about connectivity of atoms and their type. However, explicitly including these seemingly redundant features in the model can enhance performance.

In general, there seems to be a correlation between importance of features and their distribution in the dataset which can serve as a rule of thumb when selecting atomic features for new tasks. Absent features are usually ignored, and it might be profitable to exclude any features that do not appear in the dataset often enough. Information whether atom is a carbon is vital, while for other atom types, there is greater variability. The exact elements that should be encoded in a representation can be selected based on the training data. Importantly, in Section *How to find the optimal representation?*, we demonstrate that an extensive grid search over atomic features leads to poor generalization and that fixing the representation and performing only an architecture search is a very strong baseline. Finally, the results of experiments on D-MPNN indicate that rich bond representation can substitute for additional atomic features.

To the best of our knowledge, to date this is the most extensive study that focuses on the relevance of atom representation to the predictive performance of graph neural networks.

## Methods

In this section, we provide details of our experiments.

### Datasets

For evaluation, we chose four datasets that represent a range of molecular property prediction tasks. In the case of the ESOL dataset, we use two different methods of splitting the data, random split and scaffold split [66], to examine whether the choice of splitting method affects the performance of models trained with different representations. The datasets used in our experiments are:

#### Human and rat

Human and Rat are datasets for metabolic stability prediction from *Podlewska & Kafel* [24]. We use only records with the source being ’Liver’, ’Liver microsome’, or ’Liver microsomes’ resulting in 3578 (Human) and 1819 samples (Rat). In the case of multiple measurements for the same molecule, the median of the measurements is used. The stability values are expressed in hours and log scaled. 10% of the data is left out for testing, and the remaining samples are divided into 5 cross-validation folds using random stratified split.

#### QM9

QM9 is a dataset for predicting quantum properties [57]. We randomly sample 5K molecules for training, 1K molecules for validation, and 10% of the dataset (13K molecules) for the test set. The models are trained to predict g298 [free energy at 298.15 K (unit: eV)].

#### ESOL

ESOL is a water solubility prediction dataset consisting of 1128 samples [56]. We report results on both random split (from Maziarka *et al.* [17]) and scaffold split with train:validation:test ratio 80:10:10.

### Model

Graph convolutional neural networks (GCNs) are widely used models for predicting molecular properties. As input, they use molecular graphs in which vertices represent atoms and edges represent chemical bonds. To put it more precisely, a molecule with *N* atoms is represented as an undirected graph $\mathcal {G}=(X, A)$, where $X\in \mathbb {R}^{N\times D}$ is the atomic representation matrix, $A\in \mathbb {R}^{N\times N}$ is the graph adjacency matrix, and *D* is the number of atomic features. This input is processed by specialized layers, called graph convolutional layers, which are suitable for handling graph-structured data. After several graph convolutional layers, there is a pooling layer which transforms the latent representation of the input graph to a vector representation which is processed by several dense layers.

Each vertex in a molecular graph is annotated with atom features while the edges can be annotated with bond features; however, this information is optional and is not used by GCNs in our experiments. A more detailed description of the features used in molecular graphs is given in Section *Atomic and bond features*.

In this work, we use the formulation of graph convolutional layers given by Kipf and Welling [67], namely:

2 $$\begin{aligned} H^{(l+1)} = D^{-\frac{1}{2}}\hat{A}D^{-\frac{1}{2}}H^{(l)}W^{(l)}, \end{aligned}$$

where $H^{(l)}$ is the node representation matrix in the *l*-th layer, $\hat{A}=A+I$ is the graph adjacency matrix including self-loops, $D_{ii}=\sum _j \hat{A}_{ij}$, and $W^{(l)}$ is a trainable weight matrix in the *l*-th layer. The node representation at the input to the first layer is the atomic representation matrix ($H^{(0)} = X$).

In each convolutional layer, a new latent representation of the atom features is calculated and the final latent representation $H^{(L)}$ is transformed by a pooling layer. Here, we use mean global pooling:

3 $$\begin{aligned} r = \frac{1}{N}\sum \limits _{n=1}^N h_n^{(L)}, \end{aligned}$$

where $h_n^{(L)}$ is the latent representation of *n*-th atom after *L*-th convolutional layer.

In GCNs, a pooling layer has two main functions: (1) to collect information from all atoms and use it to calculate a representation for the whole molecule, and (2) to transform graph-structured representation to a vector representation suitable for dense layers.

#### Model selection

We find the best performing architectures using a random search. All neural networks consist of graph convolutional layers followed by dense layers and vary by: the number of convolutional layers, the number of channels in each convolutional layer, the number of dense layers and their size, parameters of dropout [68], presence of BatchNorm [69], the values of learning rate, batch size, and parameters of the learning rate scheduler. The number of channels in convolutional layers and the size of hidden layers are equal in all models. A detailed description of the hyperparameter space can be found in Table 9. All models are trained for 750 epochs using the Adam optimizer [70] and the MSE loss. During training, we monitor the validation loss and the final model uses weights for which this value is minimal.

**Table 9:** Hyperparameters considered in the random search and their values

Hyperparameter	Values
Number of conv. layers	1, 3, 5
Number of channels in conv. layers	16, 64, 256
Number of dense layers	1, 3
Size of dense layers	16, 64, 256
Dropout	0.0, 0.2
BatchNorm	True, false
Batch size	8, 32, 128
Learning rate	0.01, 0.001, 0.0001, 0.00001, 0.000001
Scheduler	No scheduler, decrease after 50% of epochs, decrease after 80% of epochs

We use the same set of 100 randomly sampled hyperparameter configurations for all datasets. Each architecture is trained three times to accommodate the variance resulting from random initialization. The best performing architecture is chosen based on average MSE on validation data.

#### Baseline

We compare the performance of graph-based and fingerprint-based models using as a baseline various tree ensembles trained with ECFP [19]. The length of the fingerprint representation is equal to 128 and comparable in size with the latent representation of the GCNs (see Table 9). As models, we use XGBoost [71] (XGBoost baseline) and other tree ensembles (tree-based baseline). When tuning hyperparameters of the tree-based baseline, one option is to choose an ensemble type, which can be a single decision tree, a random forest, or extremely random trees. The hyperparameters are optimized using a genetic algorithm that performs search for 24 h using 5 parallel threads. The final model is retrained on train and validation data and evaluated on test data. We use implementation from Wojtuch *et al.* [72] available in a GitHub repository (http://github.com/gmum/metstab-shap) where the detailed information about hyperparameter space and optimization procedure can be found.

#### Statistical methods

To compare atom features, we pick three best performing architectures found in random search for each representation. We perform one- and two-tailed Wilcoxon tests with Bonferroni correction to analyze the differences between representations that use different features. A single model can overfit its hyperparameters to the validation set, resulting in noisy testing set predictions. Therefore, multiple models are retrieved to reduce the influence of random weight initialization and model hyperparameters. For each compound of the test set, we calculate the median prediction of the best performing models. For the Rat and Human, the median is calculated also for all folds as these models were tuned using cross-validation. Based on these predictions, we calculate squared errors for all compounds and compare these values between representations in one-tailed Wilcoxon tests with Bonferroni correction. The alternative hypothesis is that one representation obtains lower squared prediction errors more often than the other representation.

### Feature prediction

To predict atomic features, we use a graph convolutional neural network similar to those used in other experiments. It consists of 3 graph convolutional layers (unless indicated otherwise) and for node classification we replace global average pooling and the following dense layers with 2 dense layers attached to each node (node-wise transformations). The graph layers have a hidden dimension set to 128 and dropout of 20% (probability of values being dropped). We use the Adam optimizer with learning rate $10^{-3}$ and batch size 64. The models are trained for 100 epochs. We repeat each training 5 times to reduce the impact of random initialization of the weights.

The compounds used in this experiment are randomly sampled from the ZINC database to cover a broad spectrum of feasible organic compounds. We use a random subset of 50 000 compounds which we randomly split into train and test sets using the 4:1 ratio.

### Explanations

GNNExplainer [58] is a *post-hoc* method to explain the predictions of graph convolutional neural networks. Explanations are given by a proxy model trained to minimize a mutual information objective. Given an input graph, the proxy model returns an edge mask and a feature mask, which are applied to the adjacency matrix and the feature matrix of the input graph forming a new input that maximizes the probability of a class predicted by the GCN. This new input is the explanation, and the values from the feature masks serve as feature importance scores. Note that by formulation of GNNExplainer, a single feature mask is calculated for an entire graph – as a result, the set of important features is the same for all nodes (atoms).

For each dataset and representation, we calculate graph-level explanations of predictions given by a single best-performing architecture, which gives 3 models for ESOL (scaffold), ESOL (random) and QM9, and 15 (3 runs $\times$ 5 cross-validation splits) models for Human and Rat datasets. We explain predictions for all molecules in each dataset, that is, we do not discard any molecules based on their origin (training or testing data) or any predictions based on their accuracy. In our analyzes, we only use feature masks – for each feature we obtain a single scalar value, which is the importance score.

In our experiments, we use the formulation of GNNExplainer adapted for regression implemented in PyTorch Geometric [73]. GNNExplainer is trained for 200 epochs, and other parameters have default values.

### Chemical space visualization using t-SNE

To construct a t-SNE map, we select at most 500 compounds for each representation with the highest error margin over other representations in the test set. Additionally, the error margin is averaged over top 5 models for each representation to make the resulting map less dependent on the weight initialization. Next, we calculate ECFP fingerprints to encode the chemical structure of the molecules. This representation is used as input to the t-SNE algorithm [74] along with the Tanimoto metric used to calculate distances in the fingerprint space. To find compound clusters, we use the DBSCAN clustering algorithm [75] with $\epsilon =4$. In Fig. 18, we show 5 out of 27 found clusters along with 4 compounds sampled from each of them.

## Figures and Tables

**Fig. 19:**
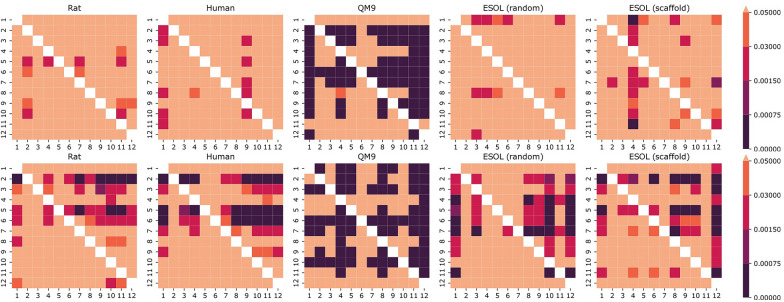
P-values of one-tailed Wilcoxon tests between the best models trained on each representation. Results for D-MPNN (top) and MAT (bottom)

## Data Availability

The code for the experiments is available online https://github.com/gmum/graph-representations. The datasets supporting the conclusions of this article are available on GitHub, http://github.com/gmum/metstab-shap (Human and Rat).

## Appendix

### Supplementary experiments using advanced graph models

To study whether our results generalize to other architectures as well, we repeat the experiments from Section *Quantitative analysis* using two advanced graph models: D-MPNN [76] which is a graph convolutional neural network capable of using molecular graphs with directed edges, and MAT [17] which is a neural network based on Transformer [14] and adapted for molecular graphs. Both models use additional information besides atomic features – MAT uses pairwise distances between atoms whereas D-MPNN uses bond features.

The test set performance of the best architectures is shown in Table 10 (D-MPNN) and Table 11 (MAT). Again, we omit variance because it is very low for all datasets but QM9. Comparing these results with those in Table 4 reveals that D-MPNN outperforms other models on three datasets – Human, QM9, and ESOL (random) – and gets close to the best model on ESOL (scaffold) which is GCN. GCNs also achieve the lowest error on the Rat dataset. Relatively poor performance of MAT might be attributed to a much smaller grid space during architecture search and possibly to a shorter training time – maximal number of epochs was equal to 100 instead of 750.^1^

**Table 10:** Average test set MSE of the best-performing D-MPNN models for each representation and dataset

Representation	Rat $\downarrow$	Human $\downarrow$	QM9 $\downarrow$	ESOL (random) $\downarrow$	ESOL (scaffold) $\downarrow$
F A	0.192 0.187	0.204 0.220	0.022 1892.635	0.113 0.117	0.181 0.193
A + N A + H A + C A + R A + A	0.189 0.191 0.193 **0.184** 0.198	0.208 0.200 0.218 **0.197** 0.214	356.488 **0.017** 0.030 1828.198 0.040	0.112 0.111 **0.100** 0.107 0.130	0.177 **0.168** 0.177 0.169 0.215
F-N F-H F-C F-R F-A	0.187 0.197 0.192 0.187 0.196	0.212 0.207 0.203 0.201 0.202	5.667 0.023 0.023 0.022 0.025	0.120 0.112 0.114 0.111 0.116	0.194 0.169 0.197 0.200 0.174

Best results are in bold

If we focus on Table 10, we might be surprised to find that all best performing models are trained with representations that only have one additional feature besides atom type. The additional feature is the inclusion in a ring for both metabolic stability datasets, the number of hydrogens in the case of QM9 and ESOL (scaffold), and the formal charge for ESOL (random). We attribute these results to the informativeness of the bond features available to the model. The performance of models trained with representations rich in information does not fall far behind the performance of models trained with information-deprived representations and in the case of ESOL (scaffold), models trained with representation without the number of hydrogens achieve error close to the best-performing model. We speculate that bond features along with atom types and one additional feature might be informative enough to ensure good performance on these datasets and that adding even more features might lead to poor generalization and overfitting, which is not unlikely because of the moderate size of the training data.

**Table 11:** Average test set MSE of the best performing MAT models for each representation and dataset

Representation	Rat $\downarrow$	Human $\downarrow$	QM9 $\downarrow$	ESOL (random) $\downarrow$	ESOL (scaffold) $\downarrow$
F A	0.205 0.325	0.241 0.260	501.171 582.430	0.135 0.246	0.214 0.483
A + N A + H A + C A + R A + A	0.235 0.231 0.317 0.233 0.237	0.258 **0.231** 0.269 0.255 0.253	569.508 559.458 583.693 517.893 570.120	0.184 0.269 0.233 0.203 0.242	0.309 0.290 0.415 0.351 0.287
F-N F-H F-C F-R F-A	0.216 **0.201** 0.204 0.202 0.207	0.240 0.254 0.240 0.241 0.239	497.775 **486.866** 497.051 544.970 488.815	0.201 0.161 **0.128** 0.200 0.136	**0.207** 0.243 0.221 0.371 0.229

Best results are in bold

The results for MAT (Table 11) differ substantially from those for D-MPNN. Here, the best performance is typically achieved by models trained on representations with more features; however, in the case of Human, the best performance is achieved by models trained with repr. A+H. It is likely that the relative positions of the atoms are not useful information and that more atom features are required to ensure good performance.

If we examine the change in performance due to adding a single feature, we see that information about neighbors proves to be the most relevant in only one case, information about hydrogens in four cases, formal charge only in one case and information about rings in three cases. Adding information about aromatic rings gives the biggest increase in performance in one case and removing it results in the second largest performance drop for D-MPNN on Rat. Removing information about rings is the most detrimental in four cases, removing information about the number of hydrogens in two cases and finally, removing information about heavy neighbors in as many as five cases. This confirms the status of the number of heavy neighbors and hydrogens as useful features, which is indicated by experiments performed on GCNs and draws attention to information about rings.

In Fig. 19, we present the results of pairwise representation comparisons with Wilcoxon tests for D-MPNN (top) and MAT (bottom). Compared to GCN, MAT exhibits stronger differences between representations, while D-MPNN seems to be less dependent on the input representation, which can be observed based on the heatmap density. The patterns visible in the figures confirm that representations A (no. 2), A+C (no. 5), A+R (no. 6), A+A (no. 7) usually lead to poorer results. The full representation (no. 1) is oftentimes a good default choice, with only a few exceptions where removing selected features (representations no. 8–12) improves model performance. Interestingly, the full representation is one of the worst choices for the D-MPNN model on the ESOL dataset, where simple representations are preferable. For MAT, the representation excluding aromaticity (no. 12) seems to be at least as efficient as the full representation across all datasets.

The results in this section demonstrate that conclusions drawn based on experiments on simple graph convolutional neural networks generalize to more advanced graph models. The importance of the number of heavy neighbors and hydrogens is confirmed, while charge and aromaticity seem superfluous. Moreover, ring information proves to be beneficial for advanced graph models. Finally, the results on D-MPNN indicate that rich bond representation can substitute for additional atomic features. On the other hand, relative positions of atoms are not useful information.

### Supplementary experiments using literature representations

The goal of this section is to put our analysis in a wider context by comparing different representations present in the literature. To this end, we repeat experiments from Section *Quantitative analysis* with four additional representations, which are summarized in Table 12. Moreover, we note that representation F (full) was used in Coley *et al.* [5]. All of these representations include the atom type and information whether it is in an aromatic system. However, the number of encoded atomic types ranges from 10 in representation F to 100 in representation Yang. All representations use an extra bit reserved for atoms that are of different type from the encoded ones. Additionally, most of the representations include information about atom’s (heavy) neighbors and formal charge.

**Table 12:** Different featurization methods used in literature

Liu [54]	
Size	Feature
23	Atom type
2	vdW radius and covalent radius
6	Is in a ring of size 3-8
1	Is in aromatic system
1	Electrostatic charge

Duvenaud [1]	
Size	Feature
44	Atom type
6	Number of neighbors
5	Number of hydrogen atoms
6	Implicit valence
1	Is in aromatic system

Li [77]	
Size	Feature
13	Atom type
6	Number of neighbors
5	Number of hydrogen atoms
6	Implicit valence
1	Is in aromatic system
1	Number of radical electrons
5	Hybridization type
1	Formal charge
1	Gasteiger partial charge

Yang [7]	
Size	Feature
101	Atom type
7	Number of bonds
6	Formal charge
5	Chirality
6	Number of hydrogen atoms
6	Hybridization type
1	Is in aromatic system
1	Atomic mass

**Table 13:** Average test set MSE of the best-performing GCN models trained with the literature representations

Representation	Rat $\downarrow$	Human $\downarrow$	QM9 $\downarrow$	ESOL (random) $\downarrow$	ESOL (scaffold) $\downarrow$
F	**0.182**	**0.218**	**9.193**	0.118	**0.166**
Liu	0.191	0.228	80.107	0.148	0.285
Li	0.223	0.223	25.155	**0.107**	0.201
Yang	0.189	0.226	24.953	0.129	0.223
Duvenaud	0.195	0.224	11.053	0.129	0.196

Best results are in bold

The averaged mean square errors are presented in Table 13. Overall, the difference in performance between the best models trained with different literature representations is usually not big. We presume that all these representations were carefully designed and are capable of achieving good performance on various tasks. However, some dissimilarities can be observed.

- Representation F outperforms all other literature representations on all datasets, but ESOL (random), even though it has the smallest size. On the contrary, representation Liu has the worst performance on all datasets but Rat.
- The differences on QM9 dataset are quite high in values which can be attributed to the range of the predicted values – QM9 models are expected to predict values in the range $(-620,-40)$, while for other datasets it is $(-4,2.2)$.
- The differences in performance are more emphasized on ESOL (scaffold) than on ESOL (random), even though the only difference between these datasets is their split. Additionally, models perform better on randomly split data. This is not surprising, as there is a greater variability between molecules with different scaffolds than between molecules that share the same scaffold. Since there is more interest in molecules from new scaffolds than from existing ones [78], the generalization capabilities become particularly important.

We conclude that a single representation can consistently outperform others on multiple tasks. Even though the differences are usually not strongly emphasized, these results indicate that comparing graph models trained with different representations is a bad practice and should be discouraged. We do not observe a correlation between representation size and model performance.

### How to find the optimal representation?

In previous sections, we demonstrated that the optimal set of features is task-specific. Here, we perform an additional analysis to answer how an optimal representation can be found – should representations be treated as another hyperparameter or maybe any representation with enough features is good enough?

We use the results of our previous experiments with GCNs and select the optimal model according to four scenarios, which vary in the amount of computational resources that they require:

- *Scenario I*—an architecture search is performed with a single predefined representation, in total $n\_architectures$ models are tested;
- *Scenario II*—first an architecture search with a predefined representation is performed to select a single best performing architecture, then this architecture is used in a representation search, in total $n\_architectures + (n\_representations -1)$ models are tested;
- *Scenario III*—just as in scenario II an architecture search with a predefined representation is performed first, but here, five best performing architectures are selected and a representation search is performed for all of them, in total $n\_architectures + 5 \times (n\_representations -1)$ models are tested;
- *Scenario IV*—representation is treated as another hyperparameter, that is, a single hyperparameter search over both architectures and representations is performed, in total $n\_architectures \times n\_representations$ are tested. We will call the model selected with this scenario an optimal solution.

**Table 14:** Mean square errors on validation and test data of models selected with different scenarios

Dataset	Scenario	I	II	III	IV
ESOL (random)	arch. repr. val. $\downarrow$ test $\downarrow$	2095 F 0.086 0.118	2095 $\underline{\textsc {F}-\textsc {A}}$ 0.083 0.120	2095 F−A 0.083 0.120	2095 F−A 0.083 0.120
ESOL (scaffold)	arch. repr. val. $\downarrow$ test $\downarrow$	3180 F 0.107 0.166	3180 F 0.107 0.166	$\underline{1990}$ $\underline{\textsc {F}-\textsc {A}}$ 0.106 0.221	1990 F−A 0.106 0.221
Rat	arch. repr. val. $\downarrow$ test $\downarrow$	996 F 0.152 0.182	996 $\underline{\textsc {A}\text {+}\textsc {H}}$ 0.147 0.196	996 A + H 0.147 0.196	996 A + H 0.147 0.196
Human	arch. repr. val. $\downarrow$ test $\downarrow$	2095 F 0.143 0.218	2095 $\underline{\textsc {F}-\textsc {C}}$ 0.141 0.213	2095 F−C 0.141 0.213	2095 F−C 0.141 0.213
QM9	arch. repr. val. $\downarrow$ test $\downarrow$	914 F 4.531 9.193	914 $\underline{\textsc {F}-\textsc {C}}$ 3.073 6.757	$\underline{917}$ F−C 2.667 9.698	917 F−C 2.667 9.698

For each model we include information about which representation (repr.) and architecture (arch.) is selected. The representation search is performed over representations 1–12. Each change in architecture or representation when choosing a more expensive scenario is underlined

In all experiments in this section, we choose the starting representation to be representation F (full) because it performs well in the previous sections and does not discard any features.

In Table 14, we show results of performing the representation search only on representations 1–12. In the case of ESOL (random), Human and Rat datasets, scenario II, which requires training only several more models than scenario I, finds the optimal solution (i.e. the same model as scenario IV). This means that scenario I, which requires testing the smallest number of models, selects the optimal architecture, but a better representation can be found. In the case of Human, increasing the number of tested models results in decreasing errors on both validation and test sets; however, for Rat and ESOL (random), the errors on test set increase, which suggests that models selected with more advanced scenarios might overfit. In the case of ESOL (scaffold) and QM9, the optimal solution is selected with scenario III, which means that the architecture selected by scenario I was not optimal but was among the top five. In the case of ESOL (scaffold), one can see that scenarios which test a higher number of models allow for a slight decrease in validation error but result in a high increase of test error—by over 30% (from 0.166 to 0.221). In the case of QM9, scenario II selects a model that outperforms the model selected with scenario I on both validation and test sets; however, the model selected with scenarios III and IV might be overfitting because the test error slightly increases (from 9.193 in scenario I to 9.698) while the validation error drops by almost 40% (from 4.531 to 2.667). In all cases, scenario I selects a model that performs similarly or better in terms of test error compared to the optimal model.

It is worth noting that in the case of Rat, all scenarios that perform a representation search select a representation that contains only information about the atom type and the number of hydrogens. For the other datasets, the chosen representations are almost full—missing only information about formal charge or aromaticity. Interestingly, representation A+H is one of two representations that are significantly inferior according to the Wilcoxon analysis in Section *Quantitative analysis* (Fig. 4).

**Table 15:** Mean square errors on validation and test data of models selected with different scenarios

Dataset	Scenario	I	II	III	IV
ESOL (random)	arch. repr. val. $\downarrow$ test $\downarrow$	2095 F 0.086 0.118	2095 $\underline{\textsc {F}-\textsc {A}}$ 0.083 0.120	$\underline{996}$ $\underline{\textsc {Li}}$ 0.078 0.126	$\underline{1743}$ Li 0.078 0.107
QM9	arch. repr. val. $\downarrow$ test $\downarrow$	914 F 4.531 9.193	914 $\underline{\textsc {Yang}}$ 2.711 14.732	$\underline{917}$ Yang 2.659 24.953	917 Yang 2.659 24.953

The representation search is performed over representations 1–12 and literature representations. Results for ESOL (scaffold), Rat and Human datasets are identical as in Table 14

In Table 15, the representation search includes the literature representations. In the case of ESOL (random), each scenario selects a different model. Scenarios II and III select models that slightly increase the test error while scenario IV selects a model that outperforms all others in terms of the test error. In the case of QM9, the optimal model is selected with scenario III; however, each time the validation error drops, there is a significant increase in the test error.

The results presented in this section demonstrate that treating representation as an additional hyperparameter can lead to overfitting. The basic scenario of fixing the representation and performing only an architecture search is a very strong baseline that in most cases gives results similar or better than when representation search is performed and several times more models are tested.

### Methods

#### D-MPNN

Directed Message Passing Neural Networks (D-MPNNs) [7] are an extension of Message Passing Neural Networks [6], which are graph convolutional neural networks, but their formulation is given from a perspective of messages sent between each pair of connected nodes and not, as is the case with GCNs, in terms of convolutional filters. In this section, we first describe Message Passing Neural Networks and then introduce D-MPNNs.

MPNNs, just as GCNs, accept as input a (molecular) graph. The initial layers of the network are responsible for calculating hidden representations of each node (atom) which is done in two phases:

1. *message-passing* – node *v* receives a message from each of its neighbors $\mathcal {N}(v)$ and aggregates all messages into a single message $m_v^{t+1}$,
2. *message-aggregation* – message $m_v^{t+1}$ is used to update hidden representation of node *v*.

Message $m_v^{t+1}$ is calculated using the current hidden representation of the node, $h_v^t$, hidden representations of all its neighbors and representations of the edges that connect the node to its neighbors:

4 $$\begin{aligned} m_v^{t+1} = \sum \limits _{w \in \mathcal {N}(v)} M_t (h_v^t, h_w^t, e_{vw}), \end{aligned}$$

where $e_{vw}$ is an edge that connects node *v* with node *w*, and $M_t$ is a message function.

A new hidden representation for node *v* is calculated using an update function $U_t$:

5 $$\begin{aligned} h_v^{t+1} = U_t(h_v^t, m_v^{t+1}). \end{aligned}$$

In the case of GCNs, these two steps are achieved using graph convolution (Eq. 2).

D-MPNNs use messages associated with *directed* edges (bonds) rather than messages associated with nodes (atoms), as is the case for MPNNs. The equation for message-passing is reformulated as follows:

6 $$\begin{aligned} m_{vw}^{t+1} = \sum \limits _{k \in \{ \mathcal {N}(v) \backslash w \}} M_t (x_v, x_k, h^t_{kv}), \end{aligned}$$

where $m_{vw}$ is the aggregated message for edge $e_{vw}$, $\mathcal {N}(v) \backslash w$ is the set of all neighbors of the starting node of edge $e_{vw}$ except for its ending node, which is *w*, $x_v$ is the representation of node *v* and $h^t_{kv}$ is a hidden representation of edge $e_{kv}$ at time *t*. Note that $h^t_{vw}$ is distinct from $h^t_{wv}$ because the edges are directed.

In our experiments, we use the implementation of D-MPNNs from https://github.com/chemprop/chemprop. We decided to closely follow the experimental setup that was used for training GCNs. We train the same number of models and use the same architectures, with one exception: the implementation of D-MPNNs, which we use, does not support BatchNorm, but introduces another important parameter, aggregation. This parameter controls the aggregation scheme, which corresponds to pooling in GCNs (Eq. 3), and can take two values in our experiments: ’sum’ or ’mean’. Since both BatchNorm and the aggregation parameter allow exactly two possibilities, we reuse the existing configuration files for GCNs and control the aggregation in D-MPNNs by the parameter that is responsible for BatchNorm in GCNs.

Importantly, all D-MPNN models additionally use bond features. First, a representation of each bond is calculated using the following features: the existence of a bond, its order, whether it is in a ring, in an aromatic ring, if it is conjugated, and 7 stereo features. Then, this representation is concatenated with the representation of a connected atom – since each bond connects two atoms, each bond has two representations, which are associated with its direction.

#### MAT

Molecule Attention Transformer (MAT) [17] is a neural network based on Transformer [14] that is adapted for molecular graphs, using their bond connectivity and 3D conformations. The main building block of MAT is its molecular attention mechanism that comprises three views of a molecule: molecular graph topology processed analogously to other graph neural networks, interatomic distances computed from the provided molecular conformation, and long-range interatomic dependencies modeled with the attention mechanism. Molecular attention is computed using the following formula:

7 $$\begin{aligned} \mathcal {A}^{(i)} = \left( \lambda _{a} \rho \left( \frac{Q_i K_i^T}{\sqrt{d_k}}\right) + \lambda _d g(D) + \lambda _g A \right) V_i, \end{aligned}$$

where $Q_i$, $K_i$, and $V_i$ are the attention queries, keys, and values calculated using input atom representations and $d_k$ is a normalization factor (defined as in Transformer [14]). *D* is the interatomic distance matrix and *A* is the adjacency matrix, $\rho$ is the softmax function, and *g* is a distance matrix transformation defined as either row-wise softmax or element-wise operation: $g(x)=e^{-x}$. $\lambda _a$, $\lambda _d$, and $\lambda _g$ are model hyperparameters such that $\lambda _a+\lambda _d+\lambda _g=1$.

In our experiments, we follow the suggestion of the authors and only optimize learning rate across 7 values (0.001, 0.0005, 0.0001, 0.00005, 0.00001, 0.000005, 0.000001). All models are trained for 100 epochs with Adam optimizer and batch size equal to 32. All MAT models use an additional feature, which is pairwise distances between all atoms.

## Abbreviations

D-MPNN: Directed message passing neural networks
GCN: Graph convolutional neural network
KRFP: Klekota-Roth fingerprint
MAE: Mean absolute error
MAT: Molecule attention transformer
MPNN: Message passing neural networks
MSE: Mean square error
QSPR: Quantitative structure–property relationship
t-SNE: t-Distributed stochastic neighbor embedding
